# Lenacapavir-induced lattice hyperstabilization is central to HIV-1 capsid failure at the nuclear pore complex and in the cytoplasm

**DOI:** 10.7554/eLife.109282

**Published:** 2026-07-08

**Authors:** Arpa Hudait, Ryan C Burdick, Ellie K Bare, Vinay K Pathak, Gregory A Voth

**Affiliations:** 1 Department of Chemistry, Chicago Center for Theoretical Chemistry, Institute for Biophysical Dynamics, and James Franck Institute, The University of Chicago Chicago United States; 2 Viral Mutation Section, HIV Dynamics and Replication Program, Center for Cancer Research National Cancer Institute at Frederick Frederick United States; Massachusetts Institute of Technology United States; Boston University United States

**Keywords:** simulation, coarse-graining, HIV, capsid, Lenacapavir, Human

## Abstract

Lenacapavir (LEN) is the first human immunodeficiency virus type 1 (HIV-1) capsid inhibitor approved for clinical use in humans. It inhibits multiple steps of the viral life cycle; however, the molecular details of the effect of LEN on capsid structure and the mechanistic steps of the inhibition are not understood. Recent studies show that intact cone-shaped capsids and capsids with LEN-induced breaks can dock at nuclear pore complexes (NPCs), but only intact capsids enter the nucleus. In this work, we combined large-scale coarse-grained molecular dynamics simulations and live-cell imaging to investigate the stepwise mechanism of docking of LEN-treated capsids into the NPC. Capsids bound to substoichiometric concentrations of LEN can reach the NPC central channel. As the capsid advances to the nuclear end, lattice defects are formed at the pentamer-hexamer interface – primarily at the narrower end – leading to pentamer dissociation. Dissociation of pentamers is detrimental to capsid integrity, leading to both rupture of the narrow end and destabilization of the hexamer-hexamer interface. Structural analysis of LEN-capsid complexes in our simulations demonstrates heterogeneous hyperstabilization and loss of the essential pliability of the capsid protein lattice. Live-cell imaging of HIV-1 cores labeled with two different fluorescent markers showed that LEN-treated ruptured capsids were docked at the NPC but were not imported into the nucleus. We conclude that LEN contributes to the loss of capsid elasticity and integrity, inhibiting HIV-1 nuclear entry and replication. Our findings demonstrate that altering viral material properties can be an effective strategy for designing human antiviral drugs.

## Introduction

After human immunodeficiency virus type 1 (HIV-1) enters the cell, several distinct steps precede the integration of the viral genome into the host genome (Müller et al., 2022; Shen et al., 2021). HIV-1 capsid consists of ~250 capsid (CA) protein hexamers and 12 pentamers to form a cone-shaped structure (Ganser et al., 1999; Li et al., 2000; Briggs et al., 2004; Gupta et al., 2023). This capsid structure is commonly referred to as an HIV-1 ‘core’. The interior of the capsid contains the viral genome and essential replicative enzymes and is the site of reverse transcription. Nuclear pore complexes (NPCs) are multiprotein assemblies embedded in the nuclear envelope that regulate nucleocytoplasmic transport of host proteins, as well as the passage of HIV-1 capsid into the nucleus (Mosalaganti et al., 2022). Early mechanistic models had proposed that viral cores uncoat in the cytoplasm or at the NPC central channel since the size of the capsid either exceeds or is comparable to the diameter of the NPC central channel (Hulme et al., 2011; Mamede et al., 2017; Burdick et al., 2017; Dharan et al., 2020). In contrast, recent studies indicate that viral cores remain intact during nuclear entry and terminally disassemble near the integration sites <1.5 hours before integration (Burdick et al., 2020; Li et al., 2021). Consistent with these results, cryo-electron tomography (cryo-ET) images have revealed that the NPC central channel dimension is amenable for docking and import of intact cone-shaped capsids into the nucleus (Zila et al., 2021; Kreysing et al., 2024). Our recent computational modeling demonstrated that the entry of intact capsids to the NPC central channel is regulated by the shape and orientation of the approach (Hudait and Voth, 2024). Specifically, cone-shaped capsids, when approaching from the narrow end, progressively dilate the central channel, which provides an energetically favorable pathway of import. The sequential steps that regulate the nuclear entry of the capsid are potential targets for CA inhibitors (Bester et al., 2020; Link et al., 2020).

Lenacapavir (LEN) is the first HIV-1 capsid inhibitor approved for clinical use in People with HIV (PWH) at both the early and late stages of HIV-1 infection (Patel et al., 2023). The cellular proteins nucleoporin (NUP) 153 and cleavage and polyadenylation specificity factor 6 (CPSF6) facilitate the transport of the capsid to the nuclear basket and then to the nuclear interior (Bejarano et al., 2019; Francis et al., 2020; Sowd et al., 2016; Chin et al., 2015). These cellular proteins are rich in phenylalanine-glycine (FG) motifs, which bind to a CA hydrophobic FG-binding pocket (Wei et al., 2022; Stacey et al., 2023), to which LEN also binds (Bester et al., 2020). At the binding pocket, LEN makes extensive contacts with adjacent CA monomer residues mediated through electrostatic and van der Waals interactions (Bester et al., 2020). Importantly, LEN is a more potent capsid inhibitor than PF3450074 (PF74), another capsid-binding inhibitor, and at much lower concentrations (Faysal et al., 2024). Recent studies have demonstrated that LEN treatment can lead to loss of capsid integrity (Faysal et al., 2024; Burdick et al., 2024). Furthermore, in the presence of LEN, there is an increase in the number of viral cores in the cytoplasm (Bester et al., 2020). This raises the question of whether LEN treatment can modulate the integrity of the capsid during nuclear entry.

The human NPC is an ~120 MDa macromolecular assembly consisting of 30 distinct NUPs (Mosalaganti et al., 2022). These NUPs form multiple heterooligomeric complexes, which assemble into a stacked outer cytoplasmic ring (CR), nuclear ring (NR), and inner ring (IR). The CR and NR consist of eight heterooligomeric Y-complex dimers arranged in a head-to-tail arrangement. The IR consists of eight spokes that are connected through linkers that provide conformational flexibility (Petrovic et al., 2022). The disordered NUPs, rich in capsid-binding FG dipeptide motifs, are also bound to the IR spokes, forming a cohesive network at the central channel (Ng et al., 2023). FG-NUPs are key to the initial docking and translocation of the viral capsid (Fu et al., 2024). The mechanism of capsid translocation driven by interaction with FG-NUPs is comparable to the transport of large cargoes mediated by transporters in the karyopherin family (Dickson et al., 2024). All-atom molecular dynamics (AA MD) simulations of membrane-embedded NPC to investigate the dynamics of capsid docking would require over a billion atoms and are computationally infeasible due to the system size and timescales of the processes of interest. Coarse-grained (CG) MD simulations, however, performed with ‘bottom-up’ CG models can afford important mechanistic insight into the dynamics of these cellular processes. ‘Bottom-up’ CG models are systematically derived from the underlying atomistic-level interactions to reproduce the molecular behavior as projected to a coarser representation (Jin et al., 2022).

In contrast, ‘top-down’ CG models have also been used for modeling nuclear entry (Kreysing et al., 2025) and capsid elasticity (Deshpande et al., 2024). While the CG simulations of the latter are argued by those authors to connect with atomic force microscopy (AFM) experiments, they do not incorporate solvent effects or the ribonucleoprotein (RNP), and they do not include LEN nor the NPC. Capsid strain and deformations have also been investigated previously using AAMD (Yu et al., 2022) with explicit IP6 and RNP. To efficiently simulate the nuclear entry of HIV-1 capsid, we developed a ‘bottom-up’ CG model (Hudait and Voth, 2024) of the human NPC and HIV-1 capsid from high-resolution cryo-ET structures (Mosalaganti et al., 2022). Our CG MD simulations of capsid docking at the NPC central channel demonstrated that lattice elasticity and the flexibility of the pore are key for the passage of the intact capsid through the channel. Importantly, the computational predictions of capsid docking to the NPC central channel have been recently validated in an HIV-1 core import at the NPC using cryo-ET (Hou et al., 2025), demonstrating how systematically derived ‘bottom-up’ CG models can accurately predict molecular details of complex biomolecular processes. To our knowledge, a comprehensive study demonstrating the stepwise details of the effects of LEN on a biologically realistic capsid (that harbors a model for the RNP) during nuclear docking (i.e. traversal from the cytoplasmic side to the central channel) has been lacking.

In this work, we investigate the docking of LEN-bound cone-shaped capsids into the NPC central channel using a combined computational and experimental approach. Our goal is to shed light on the interplay between LEN, capsid, and FG NUPs at the NPC that modulates capsid structural integrity. In our CG MD simulations, capsids bound to substoichiometric concentrations of LEN successfully dock into the NPC central channel. However, as the capsid approaches the nuclear end of the channel when translocating through the FG repeat permeability barrier, defects are formed at the hexamer-pentamer interface, predominantly at the narrow end. Computational structural analysis of LEN-bound capsid cones reveals hyperstabilization and stiffening of the hexameric lattice since LEN can only bind to the CA hexamers. The formation of the defects and lattice hyperstabilization leads to the processive loss of pentamers, followed by destabilization of the hexamer-hexamer interface, resulting in capsid rupture. We also observe a similar mechanism of stepwise rupture of the freely diffusing LEN-capsid complex, which emulates the capsid in cytoplasmic environment. Consistent with our CG MD simulations, live-cell imaging of HIV-1 cores labeled with two different fluorescent markers in cells reveals that LEN treatment caused HIV-1 cores docked at the NPC to rupture and that the broken capsids remained bound to the NPC, but the capsid contents are lost. Our results demonstrate that stress from NPC can accelerate the rupture of LEN-treated hyperstabilized capsids relative to freely diffusing capsids not docked at the NPC.

## Results

### Coarse-grained modeling and simulation

**Figure 1.**
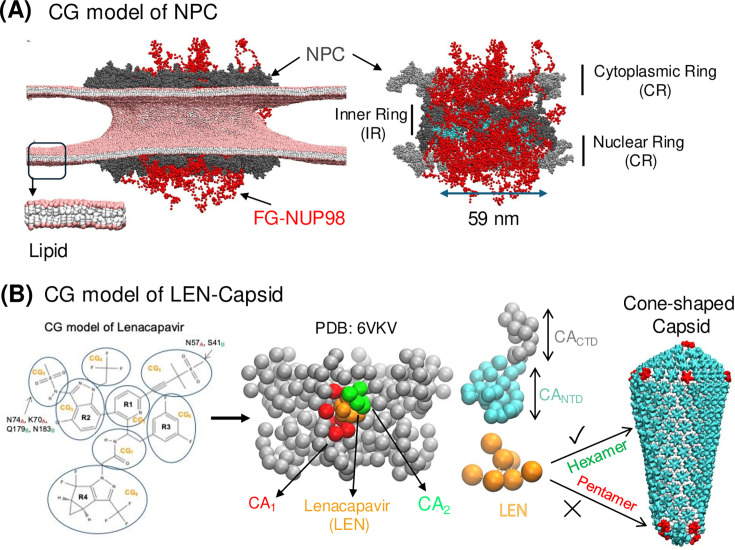
Overview of the coarse-grained (CG) molecular model of the nuclear pore complex (NPC), human immunodeficiency virus type 1 (HIV-1) capsid, and Lenacapavir (LEN). (**A**) The left panel shows the composite membrane-embedded CG model of human NPC. The NPC is shown in gray spheres. The disordered NUP98 chains are shown in red chains. The nuclear membrane is modeled with a four-site CG lipid bilayer model. The CG bead representing the lipid headgroups are shown in pink spheres, interfacial and tail beads are shown in white spheres. In the right panel, the cross-section of the NPC is shown. The cytoplasmic ring (CR) and nuclear ring (NR) are shown in silver to distinguish from the inner ring (IR) (shown in gray spheres). The FG-NUP62 is modeled as a heterotrimeric subcomplex along with NUP54 and NUP58 is shown in cyan spheres (occluded by the FG-NUP98 mesh). The diameter of the central channel is 59 nm. (**B**) The left panel shows the CG representation of LEN with CG sites 1–8 labeled on the LEN chemical representation. The center panel shows the CG-mapped representation of LEN bound to a CA hexamer. The CG-mapped structure was generated from the X-ray crystal structure PDB: 6VKV. A CG LEN molecule is shown in orange beads. The CG sites of adjoining CA monomers CA_1_ and CA_2_ that are in contact with LEN are shown in red and green, respectively. In the right panel, the CG representation of the CA monomer is shown. The CA_NTD_ and CA_CTD_ domains are shown in cyan and silver spheres, respectively. In the snapshot of the cone-shaped capsid, the CA_NTD_ of the hexamer is shown in cyan, and the pentamer is shown in red. The CA_CTD_ domain of both hexamer and pentamer is shown in silver.

We recently developed a ‘bottom-up’ solvent-free membrane-embedded CG model of the human NPC that consists of eight copies of the dimerized Y-complex and IR spokes (Hudait and Voth, 2024). The membrane-embedded composite NPC model is shown in Figure 1A. The FG-rich NUP62 is modeled as heterotrimeric subcomplex along with NUP54 and NUP58. The disordered NUP98 containing capsid-binding FG-motifs are tethered to the NPC central and create a hydrogel-like environment through multivalent interactions (Ng et al., 2023; Yu et al., 2023; Najbauer et al., 2022). We used inter-NUP98 associative interactions that result in extended conformations of the NUP98 chains, forming a mesh-like environment at the channel and closely mimicking the NPC environment in vivo (Yu et al., 2023). Importantly, we previously demonstrated that capsid docking is promoted by the extended conformations of NUP98 chains (Hudait and Voth, 2024). As the capsid translocates to the nuclear side, the conformation state of NUP98 chains in the mesh remains mostly unaltered (i.e. the extended state is preserved, Figure 1—figure supplement 1). Figure 1—video 1 depicts the capsid cone (approaching from the narrow end) docking at the NPC central channel. It should also be noted that in these and in our past CG MD simulations (Hudait and Voth, 2024) of capsid docking into the NPC, it is infrequently observed that the NPC ring can ‘break’ and the capsid docks in a somewhat sideways orientation (Figure 1—figure supplement 2). This behavior has been recently suggested experimentally as well (Kreysing et al., 2025), though we considered it to be a minor component of the ensemble of capsid-NPC docking events. We also note that in our CG MD model simulations, we do not need to apply a force to observe the capsid docking behavior (it occurs as an electrostatic ‘ratchet’), which may be contrasted with a different CG simulation approach to this docking behavior based on the top-down Martini CG model (Kreysing et al., 2025).

**Figure 1—figure supplement 1.**
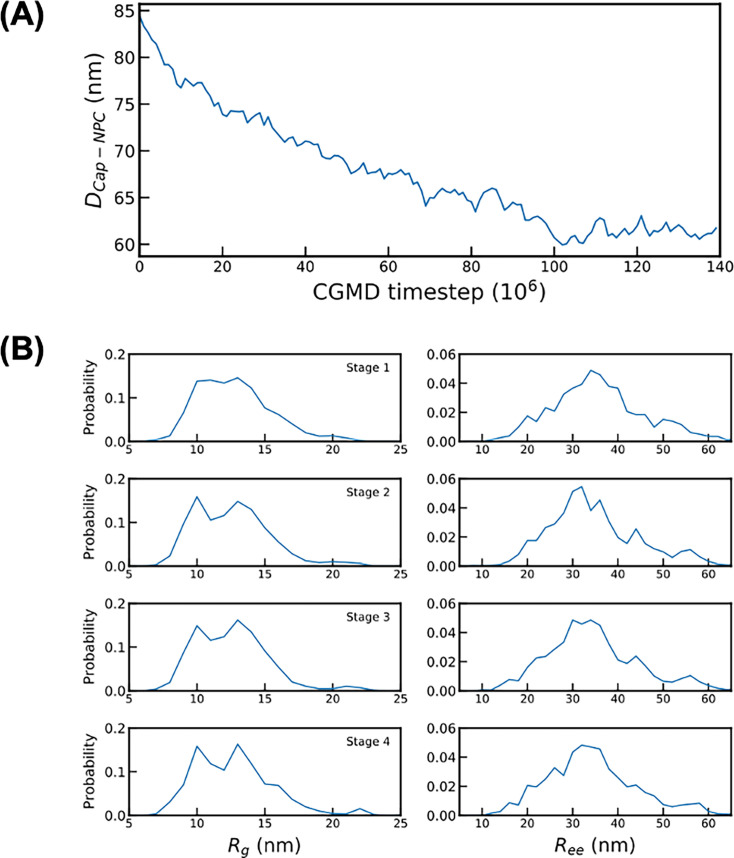
Dynamics of capsid translocation (without Lenacapavir [LEN]) to the nuclear pore complex (NPC) central channel. (**A**) The time series of the distance ($D_{Cap- NPC}$) between the geometric center of the capsid and equatorial midplane of the NPC inner ring along the channel axis. (**B**) To characterize the conformation of NUP98 chains, we characterize the probability distribution of radius of gyration ($R_{g}$) and end-to-end distance ($R_{ee}$). Stages 1–4 are defined as equally dividing the simulation time (140×10^6^ $\tau _{CG}$) into four equal segments.

**Figure 1—figure supplement 2.**
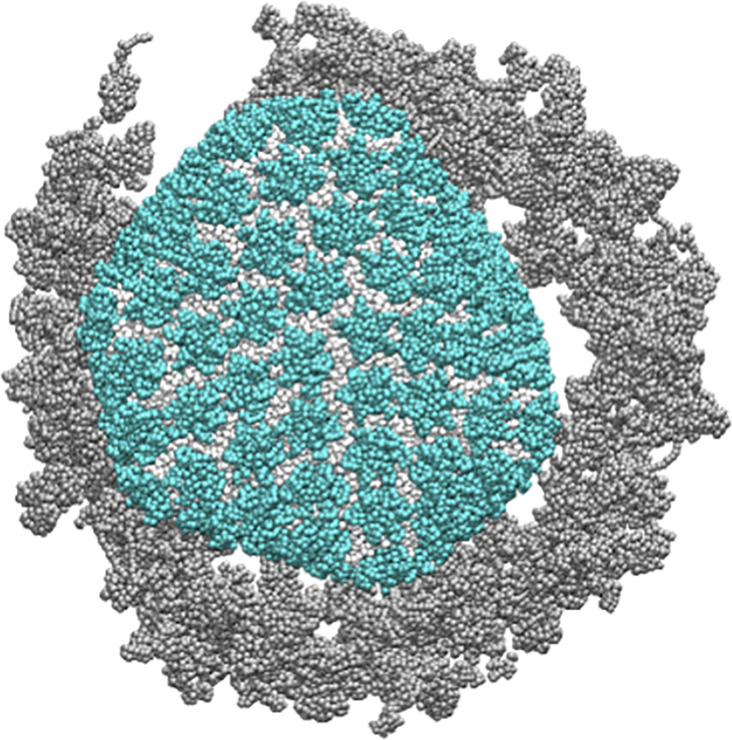
Top view of the cross-section of the nuclear pore complex (NPC) inner ring when the cone-shaped capsid is docked at the central channel and the NPC is ‘broken’. The subunits of the inner ring are shown in gray spheres. The NTD domains of both CA hexamer and pentamer are shown in cyan spheres. The CTD domain is shown in white spheres. The rest of the NPC (Y complex and NUP98 chains) and the nuclear membrane is not shown for clarity.

**Figure 1—video 1.** Docking of the cone-shaped capsid (no Lenacapavir [LEN] bound) to the nuclear pore complex (NPC) central channel. The NTD domains of CA hexamer and pentamer are shown in cyan and red spheres, respectively. The CTD domains of all CA monomers are shown in white spheres. The NPC is shown in gray, and the nuclear membrane is shown in pink (head group) and white (other groups). The rendered trajectory consists of coordinates saved every 0.5×10^6^ $\tau _{CG}$ for a cumulative simulation time of 140×10^6^ $\tau _{CG}$ (Figure 1—figure supplement 1). Initially, the NUP98 chains (shown in red) are extended. As the associative interactions between the FG-motifs of NUP98 chains and CA drive the capsid toward the nuclear end from the cytoplasmic end (via an electrostatic ‘ratchet’ mechanism), there is an increasing number of FG-CA interactions that provide the energetic driving force for capsid docking. During the docking trajectory, the NUP98 chains remain in extended conformation which continues to allow extensive FG-CA interactions, thereby providing the energetic driving force for capsid docking to the NPC central channel.

LEN (Figure 1B) is modeled with an eight-site CG model directly from the high-resolution X-ray crystal structure of LEN complexed to CA hexamer (PDB: 6VKV) (Bester et al., 2020). The CA-LEN non-bonded associative interactions (electrostatic and van der Waals) are modeled with attractive interactions of distinct energy scales. In our CG model, stronger attractive interactions are used to model the CA-LEN hydrogen bonding interactions relative to the van der Waals interactions. The magnitude of the attractive interactions was adjusted to capture the substoichiometric binding of LEN to CA hexamers (Faysal et al., 2024). We model LEN and CA interactions such that LEN molecules can only bind to CA hexamers, and all interactions to CA pentamers are turned off, as in experiments, CA selectively associates with hexamers (Stacey et al., 2023; Highland et al., 2023). We simulated LEN binding to the capsid cone (in the absence of NPC), which resulted in a substoichiometric binding (~1.5 LEN per CA hexamer), consistent with experimental data (Singh et al., 2024). Using the aforementioned CG models, we performed CG MD simulations of LEN-capsid docking at the NPC central channel. The LEN-capsid complex (bound stoichiometry of ~1.5 LEN per CA hexamer) was placed at the cytoplasmic side of the NPC, with the narrow end pointing to the central channel and touching the cytoplasmic end of the central channel. Complete details of the simulation setup are provided in the Materials and methods.

### Competition between FG-NUP98 and LEN binding regulates the early stages of the capsid docking into the NPC central channel

**Figure 2.**
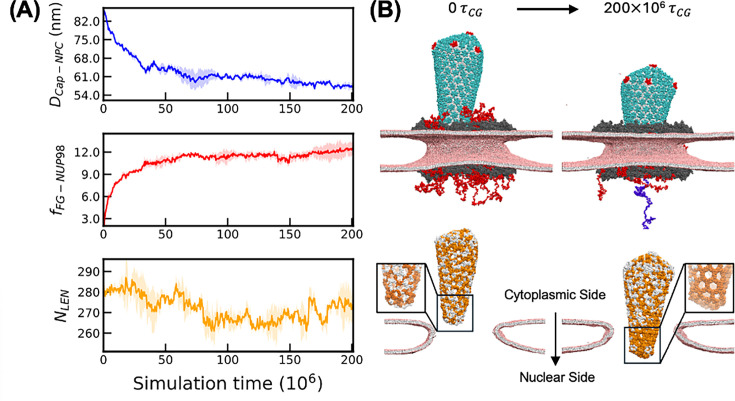
Competition between NUP98 and Lenacapavir (LEN) during docking of LEN-treated capsid. (**A**) The time series of the distance ($D_{Cap- NPC}$) between the geometric center of the LEN-bound capsid and equatorial midplane of the nuclear pore complex (NPC) inner ring along the channel axis (upper panel), the number of phenylalanine-glycine (FG) sites of NUP98 ($f_{FG- NUP98}$) directly in contact with the CA monomers (middle panel), and the number of LEN molecules bound to the capsid ($N_{LEN}$) (lower panel) are plotted. Here, $f_{FG- NUP98}$ denotes the total number of FG sites that are in the vicinity of the CA hydrophobic FG-binding pocket (within 4 nm radius). (**B**) The upper panel shows the initial configuration and the final configuration of the capsid at the end of 200×10^6^ $\tau _{CG}$. In the snapshot at 200×10^6^ $\tau _{CG}$, one ribonucleoprotein (RNP) chain (blue spheres) partly extrudes out concomitant to the first appearance of defects at the pentamer-hexamer interface. The color scheme is the same as in Figure 1. In the lower panel, only the CTD domain is shown for each CA monomer. The CA monomers to which at least one LEN molecule is bound are shown in white spheres. The CA monomers to which no LEN molecule is bound are shown in orange spheres. Note the high density of orange spheres located at the narrow end of the capsid in the right panel (docked capsid). This indicates that some LEN molecules are displaced by the NUP98 mesh to allow capsid docking. A cutaway view of the nuclear membrane is also shown to represent the degree of capsid docking from the cytoplasmic side toward the nuclear side. The NPC, LEN, and NUP98 are not shown for figure clarity. For all the plots, the solid lines are the mean values calculated from the time series of two independent replicas, and the shaded region is the standard deviation at each time step.

To simulate capsid docking into the NPC central channel, we placed LEN-treated cone-shaped capsids at the cytoplasmic end of the NPC (coplanar to the cytoplasmic Y-complex). To model the RNP complex, we added two polymeric chains in the capsid interior. The RNP complex minimally emulates the effect of nucleocapsid (NC) and RNA chains (see Materials and methods for model details). We performed two independent replicate simulations. The simulations were performed for 200×10^6^ CG MD time steps ($\tau _{CG}$=50 fs). From these simulations, to characterize the capsid docking dynamics, LEN, and NUP98 binding to the capsid, we calculated the time series statistics of three following metrics: (1) distance ($D_{Cap-NPC}$) between the geometric center of the capsid and equatorial midplane of the NPC inner ring along the channel axis; (2) the number of FG sites of NUP98 ($f_{FG- NUP98}$) directly in contact with the CA monomers; and (3) the number of LEN molecules bound to the capsid ($N_{LEN}$). The number of FG sites of NUP98 in contact with CA monomers is calculated as normalized by the number of CA monomers of all the hexamers. We plot the time series in Figure 2 of the LEN-capsid complex until the narrow end of the capsid reaches the nuclear end of the NPC, prior to the rupture events of the capsid.

**Figure 2—figure supplement 1.**
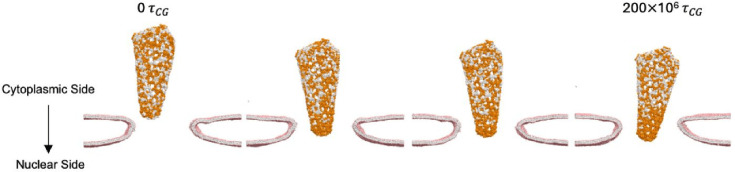
Competition between FG-NUP98 and Lenacapavir (LEN) binding during capsid translocation for Replica 2. The capsid is shown in a reduced representation (same as Figure 2). Only the CTD domain is shown for each CA monomer. The CA monomers to which at least one LEN molecule is bound are shown in white spheres. The CA monomers to which no LEN molecule is bound are shown in orange spheres. A cutaway view of the nuclear membrane is also shown to represent the degree of capsid translocation from the cytoplasmic side toward the nuclear side. The nuclear pore complexes (NPC), FG-NUP98, and LEN molecules are not shown for clarity.

Note that in this work, we use the term ‘capsid docking’ instead of ‘capsid translocation’. The process of capsid docking denotes the association of the capsid to the NPC central channel. In contrast, capsid translocation denotes the entry of the capsid into the nuclear basket, followed by the nuclear interior. The simulation of the capsid translocation to the nuclear end requires a structural model of the nuclear basket. However, when we performed this study, a structural model of the human nuclear basket was not available.

The time series profiles of docking of the LEN-capsid complex from the cytoplasmic to the nuclear side of the NPC reveal key insight into the dynamics of capsid docking (Figure 2). Initially, the LEN-capsid complex undergoes rapid passage as the number of FG sites in contact with CA steeply increases. The kinetics of passage gradually decrease as the central channel encounters the wide regions of the capsid, and there is an increasing degree of steric interaction between the capsid and the non-FG-NUPs. At this stage, the capsid is bound to the NPC mediated via the mesh-like network created by the NUP98 chains. The continuing movement of the capsid toward the nuclear end at this stage will require disruption of the FG-mesh, dissociation, and formation of FG-CA contacts. We then compared the time series of the number of LEN molecules bound to capsid and FG-CA contacts. At the late stages of LEN-capsid docking, we observe competition between LEN binding and FG-CA interactions (Figure 2B). A fraction of LEN molecules bound at the narrow end dissociate to allow NUP98 binding to the capsid (Figure 2B, Figure 2—figure supplement 1). In our simulations, LEN binding to the capsid is substoichiometric. It is possible that at significantly higher concentrations of LEN, NUP98 binding to the capsid will be adversely impacted. Therefore, our modeling demonstrates that at significantly high concentration, LEN can inhibit the efficient binding of the viral cores to the NPC, resulting in an increased number of cores in the cytoplasm.

### Distinct lattice defects at the pentamer-hexamer interface at the narrow end culminate in rupture

**Figure 3.**
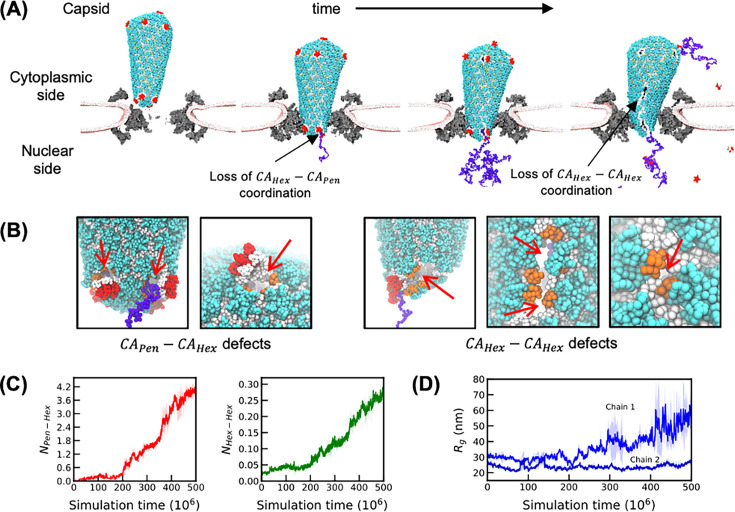
Stepwise rupture of capsid treated with Lenacapavir (LEN) during nuclear pore complex (NPC) docking. (**A**) The NTD domains of CA hexamer and pentamer are shown in cyan and red spheres, respectively. The CTD domains of all CA monomers are shown in white spheres. The ribonucleoprotein (RNP) chains are shown in blue. The NPC (cutaway view) is shown in gray, and the nuclear membrane is shown in pink (head group) and white (other groups). The initial events of CA-CA contact disruption at the hexamer-pentamer interface ($CA_{Hex}{- CA_{Pen}}$) at the narrow end are labeled with arrow. In the leftmost snapshot, the RNA extrudes out of the defect as a result of partial dissociation of the pentamer. In the final stage, nucleation of cracks occurs at the hexamer-hexamer interface ($CA_{Hex}{- CA_{Hex}}$) and extends from the narrow to the wide tip. (**B**) Snapshots of the defects at the hexamer-pentamer interface and hexamer-hexamer interface. CA_CTD_ domains of the hexamers that constitute the defect site are highlighted in dark orange spheres. Note that the CG beads constituting the CA_CTD_ domains at the defect site are disconnected from at least one nearest neighbor, leading to the locally cracked lattice (marked with red arrows). (**C**) Time series of the degree of defects at the pentamer-hexamer ($N_{Pen-Hex }$) and hexamer-hexamer ($N_{Hex-H}$). Note that $N_{Pen- }$ and $N_{Hex-Hex}$ are calculated by normalizing to the total number of CA pentamer (Burdick et al., 2020) and hexamer rings (209), respectively. The left panel shows the time series of the undercoordinated CA monomers at the pentamer-hexamer interface. The right panel shows the time series of the undercoordinated CA monomers at the hexamer-hexamer interface. (**D**) Time series of the radius of gyration ($R_{g}$) of the two RNP chains. The solid lines are the mean values calculated from the time series of two independent replicas, and the shaded region is the standard deviation at each time step.

Visualization of the NPC docking trajectories of LEN-capsid complexes revealed several key steps that lead to the loss of core integrity (Figure 3A, Figure 3—videos 1 and 2). In our simulations, pentamer-hexamer contacts are transiently disrupted, leading to dissociation of the CA pentamer rings both at the narrow and wide ends. CA pentamers at the narrow end are likely to dissociate faster than at the wide end, likely a result of weakened contacts between hexamers and pentamers due to higher curvature. Molecular fluctuations result in the loss of pentamer-hexamer contacts and lead to irreversible dissociation of the pentamers from both the narrow and the wide end (Figure 3B). Concurrent with the appearance of defects and dissociation of pentamers, we also observe RNP complex extruding through the lattice defects. Dissociation of multiple pentamers from the narrow end is detrimental to capsid integrity, resulting in lattice rupture. After rupture of the lattice, we observe the formation of defects at the hexamer-hexamer interface away from the narrow end, demonstrating further weakening of the lattice integrity. Although high computational cost precluded us from continuing these CG MD simulations, we expect these defects at the hexamer-hexamer interface to propagate from the high curvature to low curvature end of the capsid.

**Figure 3—video 1.** Docking of the cone-shaped capsid (Lenacapavir [LEN]-bound) to the nuclear pore complex (NPC) central channel (Replica 1). The NTD domains of CA hexamer and pentamer are shown in cyan and red spheres, respectively. The CTD domains of all CA monomers are shown in white spheres. The ribonucleoprotein (RNP) chains are shown in blue. LEN molecules bound to the capsid are shown in orange. LEN molecules that are not bound to the capsid are not shown for clarity. The NPC (cutaway view) is shown in gray, and the nuclear membrane is shown in pink (head group) and white (other groups). Initially, the CA-CA contacts are dissociated at the hexamer-pentamer interface, first at the narrow end and then at the wide end. The RNP complex extrudes from these defect sites. As the pentamers are dissociated, the integrity of the capsid is compromised. This leads to the formation of defects at the hexamer-hexamer interface. The movie frames correspond to snapshots every 250,000 $\tau _{CG}$.

**Figure 3—video 2.** Docking of the cone-shaped capsid (Lenacapavir [LEN]-bound) to the nuclear pore complex (NPC) central channel (Replica 2). The NTD domains of CA hexamer and pentamer are shown in cyan and red spheres, respectively. The CTD domains of all CA monomers are shown in white spheres. The ribonucleoprotein (RNP) chains are shown in blue. LEN molecules bound to the capsid are shown in orange. LEN molecules that are not bound to the capsid are not shown for clarity. The NPC (cutaway view) is shown in gray, and the nuclear membrane is shown in pink (head group) and white (other groups). Initially, the CA-CA contacts are dissociated at the hexamer-pentamer interface, first at the narrow end and then at the wide end. The RNP complex extrudes from these defect sites. As the pentamers are dissociated, the integrity of the capsid is compromised. This leads to the formation of defects at the hexamer-hexamer interface. The movie frames correspond to snapshots every 250,000 $\tau _{CG}$.

To characterize the molecular details of lattice rupture, we estimated the number of CA monomers that are undercoordinated (defined as the monomers with less than three neighbors at the trimeric CTD-CTD interface). Furthermore, we denoted defects as undercoordinated CA monomers of the hexamers at the pentamer-hexamer and hexamer-hexamer interface as $CA_{Pen}- CA_{Hex}$ and $CA_{Hex}- CA_{Hex}$, respectively (Figure 3B). In addition, we represented the defects as the number of undercoordinated CA monomers of the hexamers at the pentamer-hexamer-pentamer and hexamer-hexamer interface as $N_{Pen-Hex }$ and $N_{Hex-Hex}$ (Figure 3C). The time series profile of $N_{Pen-H}$ and $N_{Hex-Hex}$ indicates that these defects appear concurrently (~200 × 10^6^ $\tau _{CG}$). To characterize the correlation between the formation of lattice defects and RNP conformation, we calculate the radius of gyration ($R_{g}$). The time series of $R_{g}$ (Figure 3D) demonstrates that one RNP chain begins to extrude from the lattice defects at the narrow end coincident with the first appearance of a lattice defect (~200 × 10^6^ $\tau _{CG}$). The second RNP chain remained associated to the CA_CTD_ during our simulations.

To summarize, our CG MD simulations demonstrate that LEN-treated cores can efficiently dock at the NPC central channel, which is followed by the appearance of defects at the narrow and wide ends. These defects lead to dissociation of CA pentamers, crack formation at the hexamer lattice, and partial release of the RNP complex. Our results thus suggest that cores bound to substoichiometric concentrations of LEN efficiently dock at the NPC and then first rupture at the narrow end when bound to the NPC central channel.

### Ruptured LEN-viral complexes remain bound to the NPC

**Figure 4.**
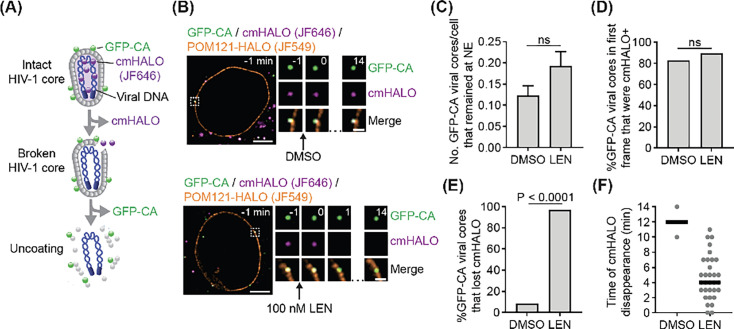
Live-cell imaging of Lenacapavir (LEN)-induced rupture of human immunodeficiency virus type 1 (HIV-1) cores docked at the nuclear envelope. (**A**) Dual-labeled viral cores: the capsid lattice is labeled with GFP-CA, and the capsid content is labeled with content marker HALO (cmHALO; JF646 dye). (**B**) Representative GFP-CA-labeled viral core in a cell expressing POM121-HALO (JF549 dye) that was stably associated with the nuclear envelope, retaining cmHALO in a DMSO-treated control cell (top) or losing cmHALO within 1 min of 100 nM LEN addition (bottom). (**C**) Number of GFP-CA-labeled viral cores per cell that remained at the nuclear envelope during the 15 min observation period. A total of 237 DMSO-treated cells and 192 LEN-treated cells were analyzed. (**D**) Percentage of GFP-CA-labeled viral cores that were cmHALO^+^ at the start of imaging. GFP-CA-labeled viral cores were analyzed for DMSO-treated (29 total) and nm LEN-treated (37 total) cells. (**E**) Percentage of GFP-CA-labeled viral cores that lost cmHALO during the 15 min observation period. (**F**) Time (min) of cmHALO disappearance following DMSO or LEN addition. p-Values were calculated using Fisher’s exact test; ns, not significant (p>0.05).

We developed an experimental live-cell imaging assay (details in Materials and methods) to assess the impact of LEN on viral cores that are stably docked at NPCs. To visualize viral cores, we labeled the capsid lattice with GFP-CA as previously described (Burdick et al., 2020) and a capsid fluid phase content with cmHALO bound to JF646 fluorescent dye, which was similar to previously described GFP content marker labeling (Li et al., 2021; Figure 4A; Figure 4—figure supplement 1, panels A–E). This dual-labeling strategy allowed us to distinguish intact GFP-CA-labeled viral cores, which retain cmHALO, from broken ones, which have lost cmHALO. To visualize NPCs, we generated a HeLa cell line stably expressing the nuclear pore protein POM121 fused to HALO, labeled with JF549 dye. Time-lapse imaging began 2 hours post-infection, with either DMSO (control) or LEN added after the first frame (Figure 4—figure supplement 1, panel F). We identified GFP-CA-labeled viral cores that remained stably docked at the nuclear envelope throughout the 15 min observation period (Figure 4B). Few viral cores remained at the nuclear envelope in either treatment condition (Figure 4C), aligning with our previous finding that most cytoplasmic viral cores fail to stably associate with the nuclear envelope (Burdick et al., 2017). At the start of imaging, most of the stably docked viral cores retained cmHALO (83–89%; Figure 4D), indicating they were intact. However, treatment with 100 nM LEN led to a rapid loss of cmHALO in nearly all viral cores compared to DMSO treatment (97% vs 8%, respectively; Figure 4E), occurring approximately 4 min after LEN addition (Figure 4F). In agreement with the CG MD simulations, these findings suggest that LEN induces rupture of NPC-docked viral cores and that the broken capsid lattice remains associated with the NPC.

**Figure 4—figure supplement 1.**
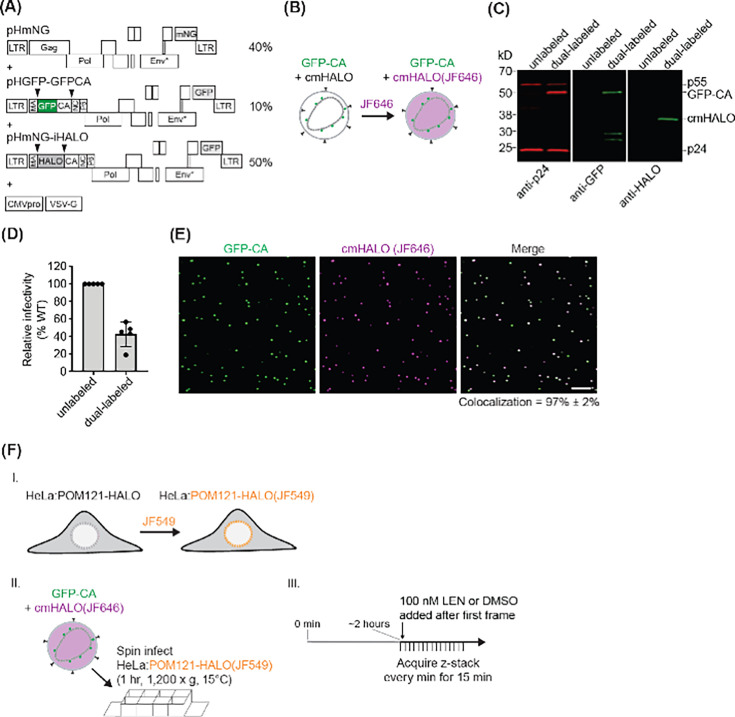
Characterization of dual-labeled human immunodeficiency virus type 1 (HIV-1) virions and live-cell imaging assay. (**A**) HIV-1 vector design for virion labeling. HIV-1 plasmids expressing WT Gag (pHmNG) or Gag with internal GFP (pHGFP-GFPCA) or internal HALO (pHmNG-iHALO) were transfected at 40%, 10%, and 50%, respectively, of the total plasmid amount. Black triangles mark protease cleavage sites. Proteolytic cleavage of Gag from pHGFP-GFPCA generates GFP-CA upon virus maturation, while cleavage of Gag from pHmNG-iHALO produces fully processed HALO protein. Asterisk denotes a mutation in the *env* gene introducing a premature stop codon; virions were pseudotyped with VSV-G. The GFP and mNG reporters in the *nef* open reading frame are expressed in virus-producing cells but not incorporated into virions. (**B**) Virions containing cmHALO were labeled with Janelia Farm JF646 dye during transfection. Excess dye was removed during virus concentration. (**C**) Western blot analysis of viral lysates comparing unlabeled and dual-labeled virions. (**D**) Effect of labeling on virus infectivity. TZM-bl cells were infected with p24-normalized amounts of unlabeled or dual-labeled virus. Luciferase activity was measured 48 hours post-infection. (**E**) Representative images of dual-labeled virions. Virions containing GFP-CA and cmHALO (JF646) were centrifuged onto a chambered slide and imaged by confocal microscopy. The percentage of GFP-CA spots that colocalize with cmHALO is shown (AVG ± SD from five images; ~80 virions/image). (**F**) Live-cell imaging of dual-labeled viral cores in HeLa cells stably expressing POM121-HALO. (**I**) HeLa cells stably expressing POM121-HALO were stained with JF549 dye for 30 min, followed by washing to remove excess dye. (II) HeLa:POM121-HALO (JF549) cells were spin-infected with dual-labeled virus. Confocal z-stacks were acquired every minute for 15 min beginning ~2 hours post-infection. Complete media containing DMSO or 2× LEN was added between the first and second frames, yielding a final LEN concentration of 100 nM.

### Rupture of LEN-treated freely diffusing capsids exhibits slower kinetics relative to NPC-docked capsids in simulations

To investigate how LEN binding modulates capsid molecular features, we simulated with CG MD freely diffusing capsid cones (not bound to the NPC), with the initial unbound concentration of LEN amounting to a stoichiometry (LEN:CA) of ~2.9:6. Here, the freely diffusing LEN-capsid complex minimally emulates the capsid at the cytoplasm. In the initial configuration, no LEN molecules were bound to the capsid. We started with a substoichiometric excess of unbound LEN molecules in the system to achieve stoichiometric saturation within the limited (relative to experiments) simulation timescales. We evolved the system for 500×10^6^ $\tau _{CG}$ and achieved an LEN-bound stoichiometry of ~1.5:6. During these simulations, CA pentamer-hexamer contacts are only transiently broken. The number of defects progressively increases, albeit at a significantly slower rate compared to the NPC-docked capsids in the same CG simulation time (Figure 5—figure supplement 1). In other words, fewer defects form in the free capsids compared to the NPC-docked capsids at similar timescales. Note that the number of LEN molecules bound to the capsid for the free capsid and NPC-docked capsids are nearly identical. Hence, the disparity in timescale of lattice rupture is not solely due to the effect of LEN on capsid lattice properties. The disparity in the timescales of the appearance of lattice defects in NPC-bound and free capsid indicates that the confining stress and steric interactions at the central channel weaken the inter-CA ring interactions of the lattice. This can effectively decrease the barrier for dissociating the pentamer-hexamer contacts and facilitate lattice rupture. As a consequence, in the CG MD without sampling (see next paragraph), we observe lattice rupture in LEN-treated capsid cones that are docked at NPCs but not when they are free in CG MD simulations.

To observe rupturing of free capsids, we performed well-tempered metadynamics (WTMetaD) simulations (Barducci et al., 2008). Briefly, WTMetaD is an accelerated sampling technique that can be used to explore high free energy barrier processes. In WTMetaD simulations, we used the mean coordination number (Figure 5—figure supplement 2) between CA proteins in pentamers and in hexamers as the reaction coordinate (WTMetaD simulation details are provided in Materials and methods). For the WTMetaD simulations, we performed four replica simulations, each with a condensed and an uncondensed RNP complex. The two interacting polymeric chains are placed inside the capsid that emulates two 9000-nt RNP complexes, and modulating the interaction strength between the CG beads of the polymers controls the condensation state of the RNP (see Materials and methods for details).

**Figure 5.**
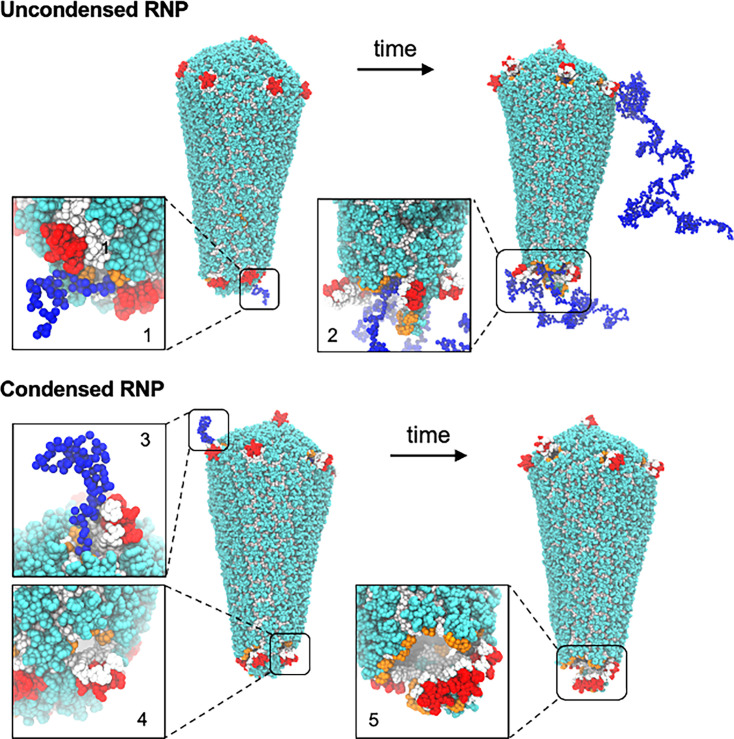
Molecular view of defects and Lenacapavir (LEN)-induced rupture of free capsids. The color scheme of the capsid and ribonucleoprotein (RNP) is the same as in Figure 3. CA_CTD_ domains of the hexamers that constitute the defect site are highlighted in orange spheres. The snapshots in the inset show a zoomed-in view of representative defects. For the uncondensed RNP, snapshot 1 shows partial dissociation of pentamers at the narrow end, and RNP chains extrude out of the capsid interior. Snapshot 2 shows a ruptured narrow end. For the condensed RNP, snapshots 3 and 4 show defects arising from the partial dissociation of pentamers at the wide end and the narrow end. Condensed RNP localizes at the capsid wide end. In snapshot 3, the RNP extrudes out of the defect at the wide end. Finally, snapshot 5 shows rupture of the narrow end.

We previously demonstrated that the RNP complex inside the capsid contributes to internal mechanical strain on the lattice driven by CA_CTD_-RNP interactions and the condensation state of the RNP complex (Hudait and Voth, 2024). The stepwise mechanism of lattice rupture for a free LEN-treated capsid observed in WTMetaD simulations is mostly similar to the NPC-bound capsid. First, pentamer-hexamer contacts are disrupted at the narrow end, followed by those at the wide end. The progressive disruption of the pentamer-hexamer contacts is followed by cracks at the hexamer-hexamer interface, leading to rupture at the narrow end (Figure 5, Figure 5—videos 1 and 2, and Figure 5—figure supplement 3). The condensation state of the RNP provides significantly different molecular environments. The uncondensed RNP model occupies the entirety of the internal space of the capsid. In contrast, the condensed RNP remains localized at the wide end, stabilized by interactions with CA_CTD_.

**Figure 5—figure supplement 1.**
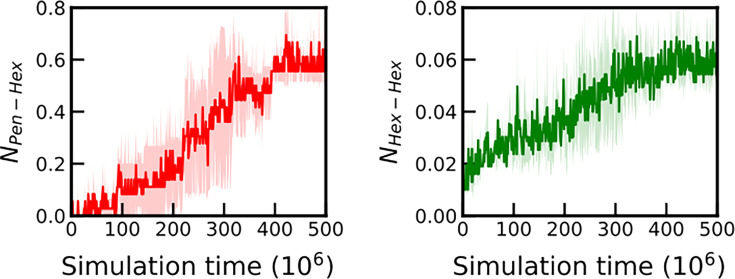
Time series of the appearance of defects at the capsid lattice in unbiased coarse-grained (CG) molecular dynamics (MD) simulations of capsid-Lenacapavir (LEN) complexes in the cytoplasm. The left panel shows the time series of the undercoordinated CA monomers at the hexamer-pentamer interface. The right panel shows the time series of the undercoordinated CA monomers at the hexamer-pentamer interface.

**Figure 5—figure supplement 2.**
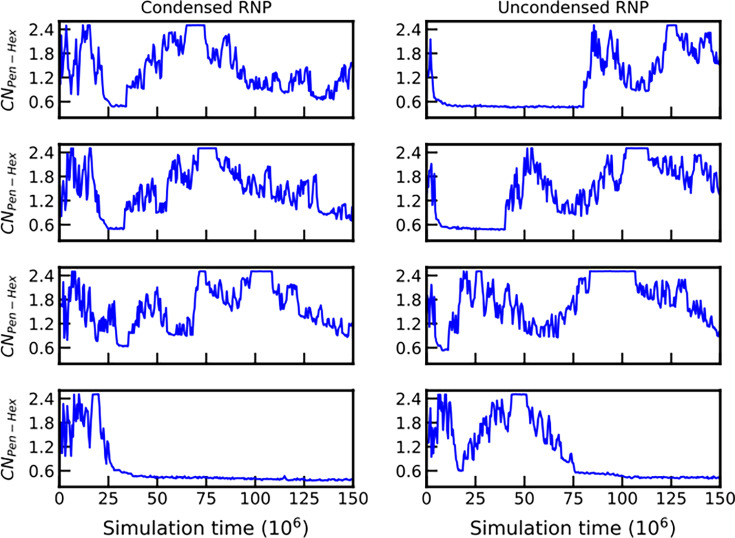
Time series of the collective variable (CV) of well-tempered metadynamics (WTMetaD) simulations of capsid-Lenacapavir (LEN) complexes in the cytoplasm. Four replica simulations (from top to bottom) are shown. The CV values fluctuate with time, indicating continuous partial dissociation (which leads to defects) and reformation of contact (healing of defects). In at least one replica (each for both uncondensed ribonucleoprotein (RNP) and condensed RNP), these defects lead to irreversible rupture of the narrow end within our simulation timescale (CV values less than 0.6).

**Figure 5—figure supplement 3.**
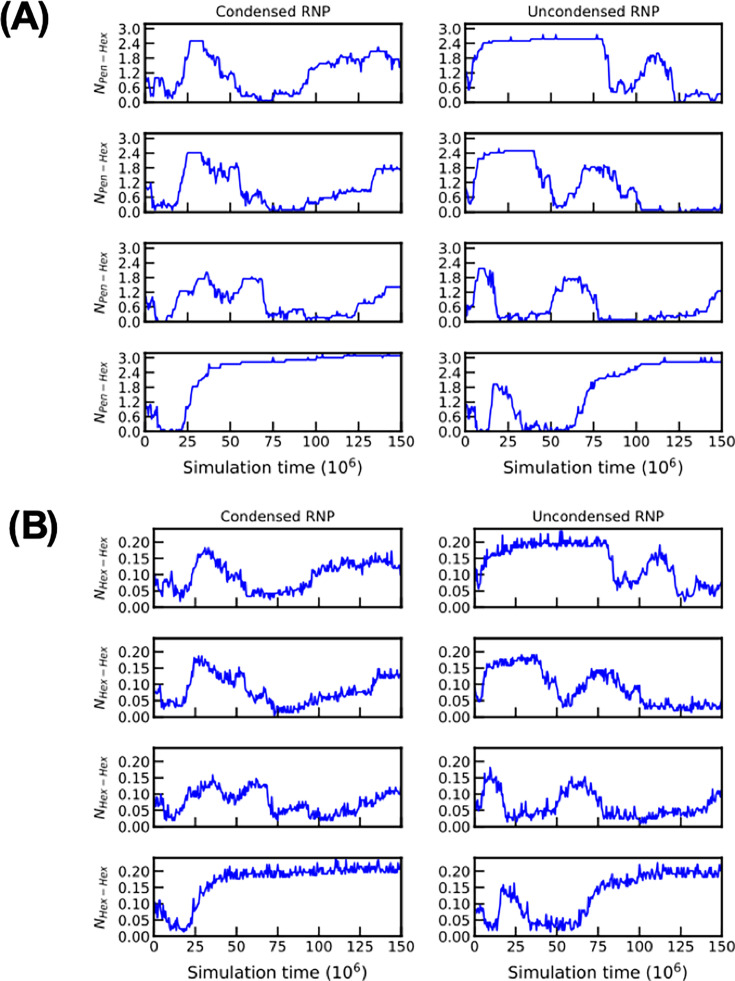
Time series of the appearance of defects at the capsid lattice in well-tempered metadynamics (WTMetaD) simulations of capsid-Lenacapavir (LEN) complexes in the cytoplasm. Four replica simulations (from top to bottom) are shown. The upper panel (**A**) shows the time series of the undercoordinated CA monomers at the hexamer-pentamer interface. The lower panel (**B**) shows the time series of the undercoordinated CA monomers at the hexamer-pentamer interface.

**Figure 5—figure supplement 4.**
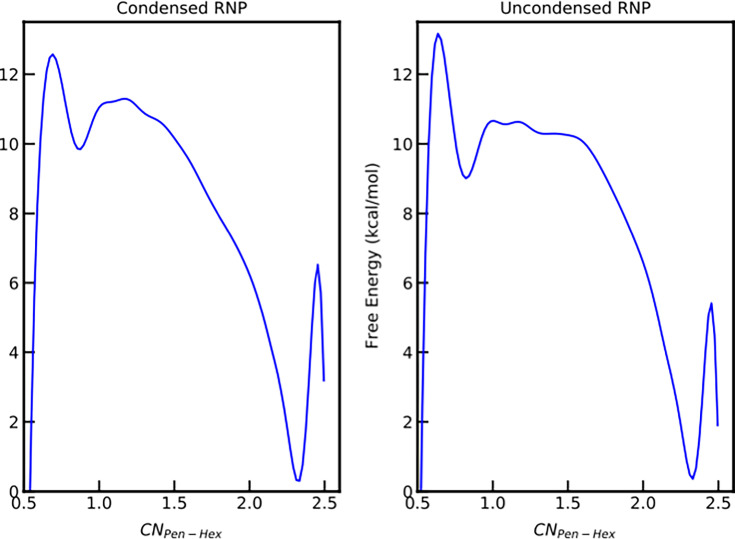
Free energy landscape of capsid disassembly of capsid-Lenacapavir (LEN) complexes in the cytoplasm. The units of the free energy are in kcal/mol. The free energy landscape is from cumulative data for all four replicas in Figure 5—figure supplement 2.

**Figure 5—video 1.** Dynamics of Lenacapavir (LEN)-induced rupture of free capsids with internal ribonucleoprotein (RNP) in condensed form. The color scheme is the same in other movies. LEN molecules that are not bound to the capsid are not shown for clarity. For ease of viewing, we aligned the capsid coordinates in the movie to the same as the initial frame, which removes the translation degree of freedom.

**Figure 5—video 2.** Dynamics of ribonucleoprotein (LEN)-induced rupture of free capsids with internal ribonucleoprotein (RNP) in uncondensed form. The color scheme is the same in other movies. LEN molecules that are not bound to the capsid are not shown for clarity. For ease of viewing, we aligned the capsid coordinates in the movie to the same as the initial frame, which removes the translation degree of freedom.

Based on the fluctuation of the reaction coordinate (mean coordination number between CA proteins in pentamers and in hexamers) in the WTMetaD simulations, we estimated the barrier of capsid disassembly to be ~11 kcal/mol (Figure 5—figure supplement 4). The free energy barrier for capsid dissociation for internal RNP in condensed and uncondensed states is nearly identical. Based on the free energy barrier of pentamer dissociation, we estimate the timescale of a single pentamer dissociation from the capsid to be ~10 μs in CG simulation time. This can rationalize why we do not observe capsid rupture in unbiased simulations in the cytoplasm, and in contrast, capsid rupture is observed during NPC docking, where the rupture is facilitated by steric effects of the NPC central channel. In a recent experiment, the timescale of capsid rupture treated with 100 nM LEN is 4 min (Li et al., 2025). A possible mechanistic pathway of capsid disassembly can be that multiple pentamers are dissociated from the capsid, and the remaining hexameric lattice remains stabilized by bound LEN molecules before the structural integrity of the remaining lattice is compromised.

Our results suggest that the condensation state of the RNP complex does not significantly impact the degree of lattice defects. This also raises the question as to whether the degree of reverse transcription in the capsid impacts the integrity of LEN-treated capsids. Both the NPC-docked and free capsid can have partially reverse-transcribed DNA in them, which likely influences their condensation state. Future studies with detailed models of RNA-DNA conversion mimicking different stages of reverse transcription could be used to investigate how DNA compaction affects the degree of lattice defects.

### LEN binding to the capsid results in hyperstabilized lattice domains

To elucidate the molecular signatures regulating the correlation between structural heterogeneity of the capsid lattice and LEN binding, we characterized the distribution of LEN bound to the freely diffusing capsid from our simulations. We found that one or two LEN molecules are associated with CA hexamers at nearly equivalent probability (Figure 6—figure supplement 1). We also observed infrequent association of three LEN molecules to the CA hexamer. This indicates that the probability of binding a third LEN molecule to a CA hexamer is impeded, likely due to steric effects that prevent the approach of an incoming molecule to a CA hexamer where two LEN molecules are already associated. Approximately 20% of CA hexamers remain unoccupied despite the availability of a large excess of unbound LEN molecules. This suggests a heterogeneity in the molecular environment of the capsid lattice for LEN binding.

**Figure 6.**
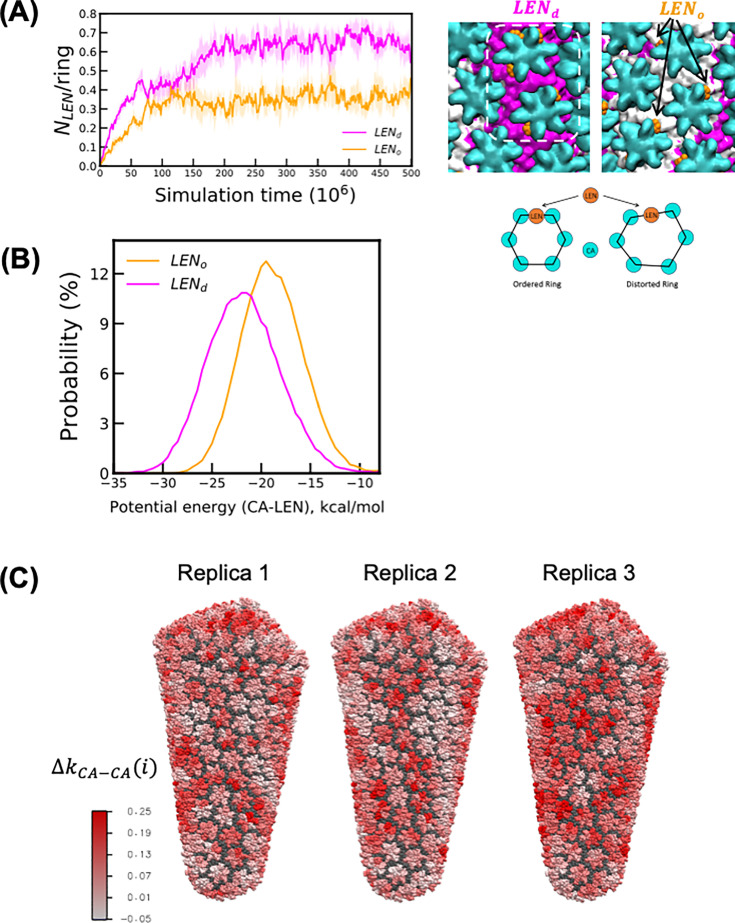
Molecular details of Lenacapavir (LEN) binding and alteration of capsid microstructure. (**A**) The left panel shows the time series of the number of LEN molecules (normalized by the number of CA hexamer rings) bound to a CA monomer which is either part of the ordered lattice ($LEN_{o}$) or distorted lattice ($LEN_{d}$). The snapshot in the left-center panel shows LEN molecules bound to regions of the lattice that are ordered. The CTD domain of the CA monomers with $\left \langle q_{6}\right \rangle _{neigh}$ < 0.4 (distorted lattice) is colored in magenta. The CTD domain of the rest of the CA monomers (ordered lattice) is colored in white. The NTD domain of the CA monomer of all the hexamers is represented as cyan spheres. The highlighted region (orange box) shows five LEN molecules bound to two adjoining CA hexamers that are classified as distorted lattice. The snapshot in the right-center panel shows LEN bound to CA that are classified as ordered lattice. The right panel shows the schematic of LEN molecule (represented as orange circle) binding to ordered and distorted CA hexamer ring. (**B**) The probability distribution of the CA-LEN potential energy calculated for all LEN molecules bound to CA sites that are part of the ordered ($LEN_{o}$) and distorted ($LEN_{d}$) lattice. The CA-LEN potential energy calculations were performed for the final 250×10^6^ $\tau _{CG}$. (**C**) Deviation of the $k_{CA- CA}\left (i\right)$ for each CA monomer $i$ relative to the value is calculated for the free capsid. The CA_NTD_ domain of all CA monomers for which the $k_{CA- CA}\left (i\right)$ increase and decrease relative to the free capsid is shown in red and blue, respectively. The red patches indicate effective stabilization relative to free capsid.

The HIV-1 capsid undergoes volume fluctuations, leading to expansive and compressive strains that manifest into highly correlated striated patterns at the capsid lattice (Hudait and Voth, 2024; Yu et al., 2022). These striated patterns also demonstrate deviations from ideal lattice packing. We characterize the distortion of the capsid lattice using neighbor-averaged Steinhardt’s local bond order parameter ($\left \langle q_{6}\right \rangle _{neigh}$) for each CA monomer (Lechner and Dellago, 2008; Steinhardt et al., 1983). Here, lower values of ($\left \langle q_{6}\right \rangle _{neigh}$) are indicative of the distortion of the lattice contiguous to a CA monomer from ideal packing. We then identified in our simulation trajectories whether a LEN molecule binds to a CA monomer that is classified as part of an ordered ($LEN_{o}$) or distorted ($LEN_{d}$) lattice (Figure 6). We found that LEN molecules bind to the distorted CA lattice sites with higher propensity than the ordered lattice (approximately 2:1), demonstrating the heterogeneous nature of the CA-LEN interactions. To rationalize this disparity in binding propensity, we calculated the distribution of the potential energy of CA-LEN interactions in the LEN-capsid complex for the LEN molecules bound to the ordered or distorted lattice sites (Figure 6). The distribution demonstrates that the binding of LEN to the distorted lattice sites is energetically favorable. Since LEN localizes at the hydrophobic pocket between two adjoining CA monomers, it is sterically favorable to accommodate the incoming molecule at a distorted lattice site. This can likely be attributed to the higher available void volume at the distorted lattice relative to an ordered lattice, the latter being tightly packed (Yu et al., 2022). This also allows the drug molecule to avoid the multitude of unfavorable CA-LEN interactions and establish the energetically favorable interactions leading to a successful binding event. Here, we denote unfavorable CA-LEN interactions as all interactions other than the electrostatic and van der Waals interactions that lead to CA-LEN binding (Bester et al., 2020). These results taken together point to the heterogeneity in the molecular environment of the capsid lattice for LEN binding.

**Figure 6—figure supplement 1.**
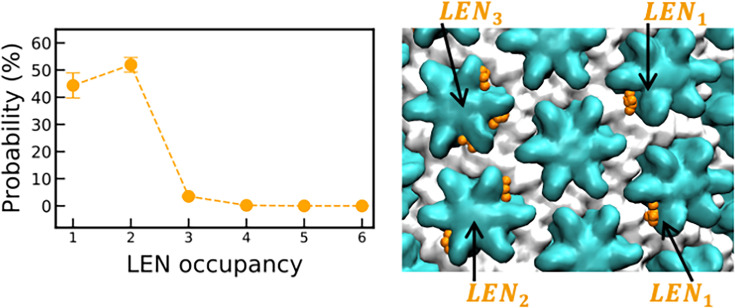
Probability distribution of the number of Lenacapavir (LEN) molecules bound to a CA hexamer. The error bars are calculated from four replica simulations. The occupancy probability distributions were performed for the final 250×10^6^ $\tau _{CG}$. The adjoining snapshot shows 1, 2, and 3 LEN molecules bound to CA hexamer labeled as LEN_1_, LEN_2_, and LEN_3_, respectively.

To determine if LEN binding modifies capsid stability, we represented the capsid as a heterogeneous elastic network model (hENM) (Lyman et al., 2008). In the hENM model, the cumulative CA inter-subunit interactions are folded into an effective spring represented by harmonic potentials (see Materials and methods for details). The effective stability (represented as spring constants) is calculated using a rigorous method by first computing the normal modes of the elastic network model. In a way, the effective spring constants calculated from the normal mode analysis are the quantification of both flexibility and stability of the +LEN capsid CG structures. Note, the elasticity defined in this work is a molecular-level property, specifically focusing on the harmonic response of CA capsomers. By focusing on the harmonic response, we analyze how these capsomer building blocks in the lattice resist stress-induced dissociation. This determines if the capsid can squeeze through the narrow NPC or if it will rupture.

A higher mean spring constant ($k_{CA- CA}$) indicates stronger effective interactions. To establish a molecular view of the effective CA inter-subunit interactions, we calculated the deviation ($\Delta k_{CA- CA} (i)$) for CA subunit ($i$). The change in $\Delta k_{CA- CA}\left (i\right)$ indicates the change in effective CA-CA interactions and illustrates how multiple CA hexamers are collectively stabilized (or destabilized) due to LEN binding (Figure 6). In the LEN-capsid complex, we observed the presence of spatially correlated CA subunits with positive $\Delta k_{CA- CA}\left (i\right)$. The size of the hyperstabilized domains (Figure 6) ranged from 80 to 130 CA subunits, illustrating that LEN binding to the capsid induces hyperstabilized domains that are distributed across the hexameric lattice. Note that multiple LEN molecules are interspersed in these domains, cooperatively inducing hyperstabilization of the lattice.

## Discussion

In this study, we combined large-scale computer simulations, live-cell imaging, and structural analysis to characterize defect formation and rupture of LEN-treated HIV-1 cone-shaped capsids that are docking into the NPC central channel. We find that the substoichiometric binding of LEN to free capsids alters the properties of the capsid, specifically hyperstabilizing and rigidifying the lattice. CG MD simulations complemented by the outcome of live-cell imaging demonstrate that LEN-treated capsids dock at the NPC and rupture at the narrow end when bound to the central channel and then remain associated with the NPC after rupture. The dynamics of lattice rupture proceed through three distinct steps: (1) disruption of CA-CA contacts at the hexamer-pentamer interface, (2) dissociation of CA pentamers at the narrow end, followed by the wide end, and (3) nucleation of cracks at the hexamer-hexamer interface, typically in the vicinity of the narrow end. The viral cores are partly ruptured but not fully disassembled. We hypothesize that LEN-treated broken cores are stabilized by the interaction with the disordered FG-NUP98 mesh at the NPC. It is noteworthy that our recent transmission electron microscopy (TEM) analysis of HIV-1 cores isolated from virions revealed that treatment of the viral cores with LEN induced a high frequency of breaks at the narrow end of the capsids (Li et al., 2025). These TEM results strongly support the CG MD simulations described in our studies – both docked at the NPC and free capsids.

The NPC allows selective and efficient transport of large cargoes between the cytoplasm and nucleus. The intact cone-shaped capsid is now understood to dock at the NPC central channel and translocate to the nuclear interior before disassembling (Burdick et al., 2020; Li et al., 2021; Zila et al., 2021). Our previous CG MD simulations of cone-shaped capsid docking into the NPC central channel demonstrated several key factors that regulate the passage of the intact capsid (Hudait and Voth, 2024). When approaching from the narrow end, there is an increasing degree of steric barrier as the central channel encounters the wider ends of the capsid. One of the several behaviors through which the capsid lattice responds to steric stress is through the formation of striated patterns of greater lattice distortion (Hudait and Voth, 2024; Yu et al., 2022). Importantly, these patterns are signatures of the lattice elasticity and a necessary form of material metastability. This structural metastability is essential in maintaining capsid integrity during nuclear entry by absorbing radially inward stress from the NPC. It is to be noted that elasticity in this context refers to a CA monomeric-level effective harmonic response and not ‘macroscopic’ elasticity. In other words, the elasticity in this work can also be interpreted as deviation of the ideal CA lattice from its ideal lattice structure in response to drug binding and confining stress. Our view is that this perspective is more useful for this problem than a macroscopic perspective as the capsid is, in fact, a mesoscopic object and not a macroscopic one. Structural analysis of LEN-treated capsids with substoichiometric concentrations of bound drug indicates the formation of hyperstabilized domains in the hexameric lattice inducing structural heterogeneity. As a consequence, the hexamer-pentamer interface is weakened relative to the hexamer-hexamer interface, resulting in the loss of pentamers, which is followed by nucleation of defects at the hexamer-hexamer interface. LEN binding stimulates similar changes in free capsids, but they occur with lower frequency on similar timescales. This suggests that the cores docked at the NPC are under increased stress, resulting in more frequent weakening of the hexamer-pentamer and hexamer-hexamer interactions, as well as more nucleation of defects at the hexamer-hexamer interface.

Once multiple pentamers are dissociated from the lattice, additional defects nucleate at hexamer-hexamer interfaces. Since all five pentamers are particularly important to maintain the high curvature of the narrow end, the loss of even a single pentamer can be detrimental to morphological integrity. The localized lattice rupture at the narrow end in our simulations with LEN is comparable with that of rupture induced by double-stranded reverse transcription products observed by AFM (Rankovic et al., 2017; Rankovic et al., 2018; Rankovic et al., 2021). As the capsid breaks at the narrow end, defects at the hexamer-hexamer interface nucleate and extend from the narrow to the wide end. Importantly, these extended defects are formed along the striated patterns of lattice disorder, which we previously identified as weak points of the capsid (Hudait and Voth, 2024; Yu et al., 2022). At the endpoint of our CG MD simulations – and consistent with live-cell imaging – although there are extended cracks and vacancy-like defects in the lattice, suggesting loss of some of the capsid lattice, the capsid does not fully disassemble and retains a large part of the hexameric lattice. This observation is consistent with the visualization of damaged capsids with significant defects (Guedán et al., 2021; Francis et al., 2022). The partly broken hexameric lattice remains bound to the FG-NUP mesh at the NPC central channel. This suggests that the alteration in the morphology of the capsid lattice induced by LEN does not impair the capsid to FG-NUP interactions, allowing stable docking of the broken cores. These observations are also consistent with our observations of frequent breaks and damage induced by LEN treatment of isolated viral cores, as well as our observations in cell-based assays indicating that broken cores can dock at the NPC (Li et al., 2025).

Multiple studies have reported two-step HIV-1 capsid uncoating: initial capsid rupture followed by terminal disassembly (Li et al., 2021; Faysal et al., 2024; Márquez et al., 2018). The uncoating of broken capsids is delayed in the presence of host factors, which likely stabilizes the lattice (Faysal et al., 2024; Burdick et al., 2024). Intrinsically disordered FG-rich NUP98 and NUP153 chains can oligomerize to higher-order condensates mediated by prion-like interactions and form a diffusion barrier for the capsid at the central channel and nuclear basket (Fu et al., 2024; Dickson et al., 2024; Milles and Lemke, 2011; Milles et al., 2013). Whether the FG-NUP hydrogel can simultaneously stabilize the broken LEN-capsid complex at the NPC and prevent nuclear import is a direction for future investigation.

To summarize, our findings using large-scale modeling with supporting live-cell imaging reveal that the capsid inhibitor LEN modulates the HIV-1 capsid metastability and lattice molecular-scale properties. We find that FG-pocket binding of LEN cooperatively hyperstabilizes the hexameric lattice, inducing structural defects and then allowing breaking of the capsid, while altering capsid integrity overall. Lattice defects arising from the loss of pentamers and cracks along the weak points of the hexameric lattice drive the rupture of the capsid. Our results suggest that in the presence of the LEN, capsid docking into the NPC central channel will increase stress, resulting in more frequent breaks in the capsid lattice compared to free capsids. LEN modulates capsid structures by generating hyperstabilized domains, leading to detrimental loss of elasticity, essential for adaptation to the crowded environment at the NPC central channel during nuclear import. These insights into the effect of LEN on capsid lattice microstructure based on our modeling and structural analysis can benefit future efforts in the rational design of more potent analogs.

## Materials and methods

### CG model of NPC

To overcome the computational cost associated with simulating the NPC in fully atomistic detail, we recently developed a CG model from available experimental structural data (Mosalaganti et al., 2022). The model is largely ‘bottom-up’, i.e., developed quantitatively from atomistic simulations of constituent NUP subcomplexes. We used a mapping resolution of ~5 amino acids per CG site or ‘bead’. The composite CG model of the NPC consists of CR, NR, and IR. The CR and NR consist of eight copies of Y-complex dimer. IR consists of eight subunits, and each subunit is composed of two copies each of NUP155, NUP93, NUP205, and NUP188. The linker NUP35 connects adjacent subunits. Two additional copies of NUP155 also connect each IR subunit to NUP160 of Y-complex of CR and NR. Our CG model also consists of the capsid-binding coiled-coil NUP62 and intrinsically disordered NUP98 chains. These proteins contain FG motifs that bind to a hydrophobic pocket in capsid (CA) monomer. NUP62 is modeled as a heterotrimeric NUP54-NUP58-NUP62 subcomplex connected using a weakly bonded harmonic network with a force constant of 0.01 kcal/mol/Å^2^ between neighboring monomers with a distance cutoff of 3 nm.

Before deriving the inter- and intra-molecular interactions of the CG proteins, each constituent NUP protein of the NPC was mapped to corresponding CG beads using the essential dynamics coarse-graining (EDCG) method (Zhang et al., 2008), in which the atomistic residues are grouped to CG beads such that the principal modes of motions sampled during atomistic simulations are preserved. In EDCG, the mapping operator (${\boldsymbol M}_{\boldsymbol {\rm R}}^{N}\colon {\boldsymbol {\rm r}}^{n}\rightarrow {\textbf {R}}^{N}$) is variationally optimized using simulated annealing to obtain the global minimum of the target residual ($\chi ^{2}$). Here, $\boldsymbol {\rm {r}}^{n}$ and $\boldsymbol {\rm {R}}^{N}$ are the configurations of the atomistic and CG trajectories, respectively. The all-atom to CG mapping function is adjusted during the optimization to minimize the target residual ($\chi ^{2}$):

(1) $$\displaystyle  \chi ^{2}=\frac{1}{3N}\sum _{I=1}^{N}\left \langle \sum _{i,j}|r_{i}- r_{j}|^{2}\right \rangle _{t}\colon i,j\epsilon I,j\geq i$$

where $N$ is the total number of CG sites. Here, $i,j$ are the unique pairs in the group of all-atom residues that are part of the CG site, $I$. $\boldsymbol {r}_{i}=\boldsymbol {x}_\boldsymbol {i}- \left \langle \boldsymbol x_{\boldsymbol i}\right \rangle _{t}$ is the displacement of atom $i$ from the atom’s mean position, $\left \langle \boldsymbol x_{i}\right \rangle _{t}$. The residual ($\chi ^{2}$) is small when the atoms $i,j$ move in a correlated fashion, i.e., the displacements $\boldsymbol r_{i}$ and $\boldsymbol r_{j}$ are similar.

By design, it is easier to minimize the residual ($\chi ^{2}$), if a single residue in atomistic resolution is mapped to one CG bead (i.e. 1→1 mapping), and reverse when very coarse mapping is used. However, computational cost also increases for finer CG models. Therefore, our choice of the mapping resolution (i.e. average number of residues per CG beads) is based on an empirical ‘elbow rule’, i.e., the $\chi ^{2}$ value with increasing value of $N$, the $\chi ^{2}$ value decreases and reaches a plateau. We chose the $N$ value at the elbow point, at which $\chi ^{2}$ reaches the plateau.

The intermolecular non-bonded CG interactions between NUP monomers and subcomplexes were modeled using repulsive excluded volume and attractive interactions. The repulsive excluded volume interactions were used to prevent overlap between CG beads. The repulsive excluded volume interaction $(E_{excl})$ was modeled with a soft cosine potential,

(2) $$\displaystyle E_{excl}\left (r_{ij}\right)=A\left (1+\mathrm{cos}\left (\frac{\pi r_{ij}}{r_{c}}\right)\right)$$

where $r_{ij}$ is the pairwise distance between CG site types *i* and *j*. $A$ is 15 kcal/mol for all $ij$ pairs. The distance cutoff ($r_{c}$) for the excluded volume interactions was 1.0 nm. The non-bonded attractive interactions between NUP subcomplexes at the key binding interactions were modeled with pairwise Gaussian potential ($E_{gauss}$),

(3) $$\displaystyle E_{gauss}\left (r_{ij}\right)=\frac{H_{ij}}{\sigma _{ij}\sqrt{2\pi }}\mathrm{exp}\left (\frac{- \left (r_{ij}- r_{0,ij}\right)^{2}}{2\sigma _{ij}^{2}}\right)$$

where $r_{0,ij}$ and $\sigma _{ij}$ are the mean and standard deviation of the distance between CG site types $i$ and $j$, respectively. For all CG $ij$ pairs $\sigma _{ij}$ value of 0.12 nm was used for all non-bonded attractive interactions modeled with $E_{gauss}$. The distance cutoff of 3 nm is used for all. The constant $H_{ij}$ for each binding interface was optimized using Relative Entropy Minimization from the atomistic trajectories of the respective all-atom subcomplexes (Shell, 2008).

CG NUP98_1-620_ chains consist of the structured GLEB domain (residues 157–213) and the intrinsically disordered region (residues 1–156 and residues 214–620) modeled as a linear polymer chain. Each NUP98_1-620_ consists of 124 CG beads. Each CG bead of the polymer chain is linked with a harmonic spring with an equilibrium bond length of 2 nm and a force constant of 0.5 kcal/mol Å^−2^. The intrinsically disordered regions (residues 1–156 and residues 214–480) predominantly consist of the capsid-binding FG-motif. The intra-chain and inter-chain interactions between CG beads of the FG-rich region (residues 1–156 and residues 214–480) were modeled using a 12-6 Lennard-Jones potential ($E_{scLJ}$) with a modified soft-core (Beutler et al., 1994):

(4) $$\displaystyle E_{scLJ}\left (r\right)=4\varepsilon \lambda ^{n}\left [1/\left (\alpha _{LJ}\left (1- \lambda \right)^{2}+\left (r/\sigma \right)^{6}\right)^{2}- 1/\left (\alpha _{LJ}\left (1- \lambda \right)^{2}+\left (r/\sigma \right)^{6}\right)\right ]$$

where $n$ = 2, $\alpha _{LJ}$ = 0.5, $\alpha $ = 0.6, and $\sigma $ = 1.25 nm. The distance cutoff of 3 nm was used. The strength ($\varepsilon $) of the inter- and intra-chain interactions used was 0.3 kcal/mol. A total of 48 NUP98 chains tethered to NUP155 proteins at the IR.

The protocol to create and equilibrate the composite NPC-membrane system is described in our previous publication (Hudait and Voth, 2024). The dimension of the lipid bilayer in the $x$ and $y$ direction is 200 nm. The CG lipid was modeled using a four-site model consisting of one head, one interfacial, and two hydrophobic tail beads using the same potential energy function as in Grime and Madsen, 2019. Membrane-binding interactions of NPC are modeled with $E_{scLJ}$. Here, $n$ = 2, $\alpha _{LJ}$ = 0.5, $\alpha $ = 0.6, and $\sigma $ = 1.5 nm. The distance cutoff of 4 nm was used. Protein-lipid interactions were added between the CG sites of the β-propeller domain of NUP155, NUP133, and NUP160, and the CG lipid head group.

### CG model of HIV-1 capsid and RNP

The CG model of HIV-1 capsid was derived from an atomistic simulation trajectory of a composite system of three CA hexamers complexed with IP6 and FG peptides. The details of the atomistic simulation and CG model development protocol are described in Hudait and Voth, 2024. Each CA monomer consists of 46 CG sites with a mapping resolution of ∼5 AA residues per CG site. The intramonomer bonding topology of the CA monomer is modeled as a heterogeneous elastic network with a radial cutoff of 3 nm. The non-bonded interactions between CA monomers are modeled with a combination of repulsive excluded volume ($E_{excl}$) and attractive pairwise Gaussian interactions ($E_{gauss}$). The value of *A* in $E_{excl}$ was 15 kcal/mol for all $ij$ pairs of CA. The distance cutoff ($r_{c}$) for the excluded volume interactions was set at 1.0 nm. The parameters $r_{0,ij}$ and $\sigma _{ij}$ of $E_{gauss}$ were determined from the atomistic simulation trajectory. The constant $H_{ij}$ for all CG $ij$ pairs was optimized using Relative Entropy Minimization from the corresponding atomistic simulation trajectory (Shell, 2008). The parameters of $E_{gauss}$ for the non-bonded attractive interactions between CA and the FG peptide were derived using the identical protocol as CA-CA interactions.

The RNP complex within the capsid is modeled as a linear polymer chain containing 3000 beads. In our simulations, there are RNP chains. The non-bonded interactions between CA monomers are modeled with $E_{scLJ}$. Here, $n$ = 2, $\alpha _{LJ}$ = 0.5, $\alpha $ = 0.6, and $\sigma $ = 1.25 nm. The condensation state of RNP was regulated by varying the strength of the RNP-RNP interaction ($\varepsilon _{RNP}$). To model uncondensed RNP and condensed RNP, $\varepsilon _{RNP}$ values of 0.3 kcal/mol and 0.7 kcal/mol were used. To emulate electrostatic interactions between RNP and the basic residues at the C-terminal of CA, we used attractive pairwise Gaussian interactions ($E_{gauss}$) between the RNP and charged residues in the C-terminal end of CA ($H_{ij}$ = –4.5 kcal/mol, $r_{0,ij}$ = 1.25 nm, and $\sigma _{ij}$ = 0.12 nm).

### CG model of LEN

The CG model of LEN and LEN-CA molecular interactions was derived directly from the experimental X-ray crystal structure of six LEN molecules complexed to a CA hexamer (PDB: 6VKV) (Bester et al., 2020). The CG LEN model consists of eight sites: (1) CG_1_ – pyridinium ring (R1), (2) CG_2_ – 2-(methanesulfonyl)-2-methylpropane group attached to the pyridinium ring (R1), (3) CG_3_ – indazole ring (R2), (4) CG_4_ – trifluoroethyl (-CH_2_-CF_3_) group attached to the indazole ring (R2), (5) CG_5_ – sulfonamide group attached to the indazole ring (R2), (6) CG_6_ – difluorobenzyl ring (R3), (7) CG_7_ – acetamide group attached to the pyridinium ring (R1), (8) CG_8_ – cyclo-pentapyrazole ring (R4). The CG LEN is modeled as a rigid body, i.e., without any intramolecular fluctuations. LEN interacts with two adjoining CA monomers at the binding pocket through hydrogen bonding and van der Waals interactions (Bester et al., 2020). Specifically, all the ring systems of LEN interact with multiple residues of CA_1_-NTD and CA_2_-CTD through extensive van der Waals interactions. Additionally, LEN forms hydrogen bonding interactions with N57, K70, and N74 of CA_1_-NTD, S41 of CA_2_-NTD, and Q179 and N183 of CA_2_-CTD. Specifically, the sulfonyl group of the R1 ring and the sulfonamide group of the R2 ring form the hydrogen-bonding network of interactions with the CA subunits.

Note, our decision to derive the non-bonded interactions of the LEN CG model directly from the experimental crystal structure is due to the lack of a reliable atomistic force field. Specifically, small changes in the force field parameters of the electrostatic groups of LEN lead to unrealistic variation in the affinity or dissociation free energy of the drug molecule to CA due to the lack of a reliable atomistic force field. Lack of a reliable atomistic force field is also a barrier in deriving reliable intramolecular CG topology (bonds, angles, and harmonic force constants).

The CG interactions between CA and LEN were modeled using pairwise repulsive excluded volume ($E_{Excl}$) and attractive ($E_{Gauss}$) interactions. The distance cutoff ($r_{c}$) for the excluded volume interactions between CA-LEN and LEN-LEN interactions was set at 1.25 nm. The value of $A$ was set to 15 kcal/mol for all $ij$ pairs. In our CG model, the LEN-CA associative interactions are modeled using two different energy scales to model the hydrogen bonding ($H_{ij,HB}$) and van der Waals interactions ($H_{ij,VDW}$). First, we mapped the six LEN molecules complexed to a CA hexamer (PDB: 6VKV) from atomistic to CG resolution. We first assigned $H_{ij,VDW}$ between LEN CG beads and CA CG beads to mimic the extensive van der Waals interactions between LEN ring systems and CA residues. Then we assigned $H_{ij,HB}$ between LEN CG beads and CA CG beads to mimic the hydrogen-bonded interactions between LEN sulfonyl (R1) and sulfonamide (R2) groups and CA residues. The $r_{0,ij}$ values of the LEN-CA CG interactions were derived directly from the CG-mapped structure of six LEN molecules complexed to CA hexamer. In the simulations, $H_{ij,VDW}$ is chosen (–6.25 kcal/mol/nm) such that when $H_{ij,HB}$ is turned off, multiple LEN molecules continuously associate and dissociate at the CA hexamer binding pocket. In other words, in the CG LEN model, the energy scale of the van der Waals interactions is not strong enough to initiate the association of LEN to the capsid. The value of $H_{ij,HB}$ (–8.50 kcal/mol/nm) is the minimum CA-LEN binding strength required to initiate LEN association. Finally, in our simulations, all attractive interactions between LEN and CA monomers that are part of the pentamer ring are turned off, as in experiments, LEN selectively associates to CA hexamers (Highland et al., 2023).

### CG simulations

All CG MD simulations were performed using the large-scale atomic/molecular massively parallel simulator (LAMMPS) (Plimpton, 1995). Additional details of the CG models of different components (NPC, lipid, HIV-1 capsid and RNP) are described in Hudait and Voth, 2024. The details of the construction of the composite membrane-embedded NPC are provided in Hudait and Voth, 2024. The CG MD simulations were performed using time step ($\tau _{CG}$) 50 fs. The equations of motion were integrated with the Velocity Verlet algorithm, and the simulation setup was periodic in all ($x,y,z$) dimensions. In the initial system, the cone-shaped LEN-capsid complex was placed such that the narrow end is coplanar to the cytoplasmic Y-complex, and the axis of the cone is the same as the $z$ axis. Note the $\left (x,y\right)$ plane is the plane of the nuclear membrane. An additional 800 LEN molecules are randomly placed in the void region of the simulation cell, specifically above the nuclear membrane, both at the cytoplasmic and nuclear sides. The production simulations of the NPC central channel were performed in the constant $Np_{xy}T$ ensemble at 310 K and 1 bar. The temperature of the system was maintained using a Langevin thermostat with a coupling constant of 2000 $\tau _{CG}$ (Schneider and Stoll, 1978), and pressure was maintained using a Nose-Hoover barostat with a coupling constant of 4000 $\tau _{CG}$ (Martyna et al., 1994). In the CG simulations, LEN is modeled as a rigid body. Therefore, the translational and rotational motions of LEN molecules are modeled using rigid body dynamics (Martyna et al., 1992; Martyna et al., 1996).

The non-NPC simulations of LEN binding to the capsid were performed in a simulation cell of dimension 300 nm in $x$, $y$, and $z$ directions. The periodic boundary condition is used in all directions. Initially, the capsid is positioned such that the geometric center of the capsid is the same as the center of the simulation box. 600 LEN molecules were initially randomly positioned in the void space of the simulation cell, such that LEN molecules are at least 2 nm away from the capsid surface. The simulations were performed at 310 K, and the temperature was maintained using a Langevin thermostat with a coupling constant of 1000 $\tau _{CG}$ (Schneider and Stoll, 1978). Simulation trajectory snapshots were saved every 50,000 $\tau _{CG}$.

We performed WTMetaD simulations to facilitate the rupture of HIV-1 in non-NPC simulations (Barducci et al., 2008). These simulations mimic in vitro conditions. To define the collective variable (CV) for WTMetaD simulation, we calculated the number of CA monomers in hexamer rings ($CA_{hex}$) that are direct neighbors of the CA monomers in pentamer rings ($CA_{pen}$). To this end, we calculate the geometric center of the CG beads of the C-terminal domain. The number of adjacent $CA_{he}$ for each $CA_{pen}$ was calculated with a radial cutoff of 4.0 nm, considering the geometric center of the CTD of each CA monomer. Then, we calculate the mean value of $CA_{pen}$ and apply bias to this CV ($CN_{Pen-Hex }$). The mean coordination number function decays to zero between 4.0 nm and 4.5 nm. The time series of the CV values are shown in Figure 5—figure supplement 2. For computational efficiency, we set the upper limit of CV, a weak harmonic restraint of force constant 10,000 kcal/mol/nm^2^ to prevent the CV value from exceeding 2.5. In other words, this harmonic restraint is necessary to prevent over-coordination of CTD domains. The height of Gaussian biases was set to 0.6 kcal/mol and was deposited every 500 $\tau _{CG}$ with a bias factor of 100 and width of 0.1.

The trajectories of the CG MD simulations were visualized, and the simulation snapshots were prepared in Visual Molecular Dynamics (VMD) software (Humphrey et al., 1996).

### Capsid-LEN binding analysis

To determine if a LEN molecule is bound to CA monomers in the simulations, we used the following criteria:

(5) $$\displaystyle  d_{CA- LEN}=\left|\frac{1}{N_{CA}}\sum _{i=1}^{N}r_{CA\left (i\right)}- \frac{1}{N_{LEN}}\sum _{i=1}^{N}r_{LEN\left (i\right)}\right| $$

where $d_{CA- LEN}$ is the distance between the center of mass of the selected CG sites of LEN and CA. $r$ is the coordinate of the selected CG sites of CA and LEN. For the calculation of the center of mass of LEN, the CG beads 1, 3, 6, and 8 are used, which correspond to the ring systems R1, R2, R3, and R4. For the calculation of the center of mass of CA, the CG beads 8 (residues 34–40), 10–13 (residues 48–76), 21–22 (all-atom residues 99–105), 34–37 (residues 170–182) were used. A CA monomer and LEN molecule are in contact if $d_{CA- LEN} < 3\, \rm nm$. To determine $N_{bound}$ (number of LEN molecules bound to CA hexamers), we only consider LEN molecules that simultaneously contact two adjoining CA monomers of a hexamer.

### Capsid lattice order analysis

To determine the lattice order of the capsid, specifically whether constituent CA monomers of a ring are geometrically ideal or distorted, we used the neighbor averaged ($\left \langle q_{6}\right \rangle _{neigh}$) Steinhardt bond order parameter (Lechner and Dellago, 2008; Steinhardt et al., 1983). First, we created a reduced form of the CG MD simulation trajectories by selecting the CG site 21 (residues 99–105) of each CA monomer of the capsid. We note that CG site 21 is approximately the geometric center of a CA monomer. Therefore, in the reduced form, the capsid consists of 1314 beads, which is the same as the number of CA monomers. Using the reduced form of the trajectory, the neighbor averaged Steinhardt bond order parameter of a CA monomer is calculated as follows:

(6) $$\displaystyle  \left \langle q_{6}\right \rangle _{neigh}=\left (\frac{4\pi }{2l+1}\sum _{m=- l}^{l}\left|\bar{q}_{lm}\right| ^{2}\right)^{1/2}$$

where $\bar{q}_{lm}\left (i\right)=\frac{1}{N_{B}\left (i\right)}\sum _{k=0}^{N_{B}\left (i\right)}q_{lm}\left (i\right)$ and $l=6$. Here, $N_{B}$ is the number of neighbors of CA monomer $i$, and the reference CA monomer itself. The term $q_{lm}=\frac{1}{N_{B}}\sum _{i=1}^{N_{B}}\Upsilon _{lm}\left (\theta _{i}\left (r\right),\phi _{i}\left (r\right)\right)$, and $\Upsilon _{lm}\left (\theta _{i}\left (r\right),\phi _{i}\left (r\right)\right)$ are spherical harmonics of rank *l* and *m*, where *θ_i_* (*r*) and *φ_i_* (*r*) are the polar angles of each of the *N_B_* bonds between the central CA monomer and neighboring CA monomers. From a central CA monomer *i*, we consider the three closest CA neighbors. We defined a CA monomer to be in a geometrically ordered environment if $\left\langle q_{6}\right\rangle _{nei} > 0.4 $.

### Capsid lattice elasticity analysis

To evaluate how LEN binding modulates capsid stiffness, we used the hENM method (Lyman et al., 2008). In the hENM framework, harmonic bonds are assigned during the analysis to all CG site pairs *ij* within a cutoff distance from the central CG site $i$. The harmonic force constants (*k_ij_*) for each bond have the same value, which are then iteratively optimized by computing the normal modes of the elastic network model, solving the eigenvalue problem,

(7) $$\displaystyle \boldsymbol {H}\boldsymbol{v}_{\boldsymbol{k}}=\omega _{k}^{2}\boldsymbol {Mv}_\boldsymbol {k}$$

where $H$ is the Hessian: $H_{i,j}=\left.\frac{\partial ^{2}V}{\partial q_{i}\partial q_{j}}\right|_{m} $, $M$ is the diagonal matrix for the masses of the particles, and $\omega _{k}$ frequency for the mode of motion. The solution to the equation of motion

(8) $$\displaystyle \boldsymbol {M}\frac{d^{2} \boldsymbol {q}}{dt^{2}}+\boldsymbol {Hq}=0$$

where ***q*** is the generalized coordinate, and that for $N$ classically, interacting particles near the potential energy minimum, $\boldsymbol q_{m}$:

(9) $$\displaystyle  V\left (\boldsymbol {q}\right)=V\left (\boldsymbol q_{m}\right)+\left.\sum _{i}\frac{\partial V}{\partial q_{i}}\right| _{m}\left (q_{i}- q_{i,m}\right)+\frac{1}{2}\left.\sum _{i,j}\frac{\partial ^{2}V}{\partial q_{i}\partial q_{j}}\right|_{m}\left (q_{i}- q_{i,m}\right)\left (q_{j}- q_{j,m}\right)+O\left (\boldsymbol {q}- \boldsymbol {q}_{m}\right)^{3}$$

$V\left (\boldsymbol {q}_{m}\right)$ is a constant, and $\left.\frac{\partial V}{\partial q_{i}}\right| _{m}$ is zero. Using the normal modes, the amplitudes are then scaled according to equipartition energy that reflects the temperature of the reference simulations. The harmonic force constants ($k_{ij}$) are then iteratively optimized so that mean-squared fluctuations of the coarsened system $\left(\left \langle r_{ij}^{2}\right \rangle =\left \langle \left (x_{ij}- \left \langle x_{ij}\right \rangle \right)^{2}\right \rangle \right) $ match that of the fine-grained reference system

(10) $$\displaystyle \frac{1}{k_{ij}^{n+1}}=\frac{1}{k_{ij}^{n}}- \alpha \left (\left \langle r_{ij}^{2}\right \rangle _{CG}- \left \langle r_{ij}^{2}\right \rangle _{ref}\right)$$

Here, $n$ is the number of iterations and $\alpha $ is a parameter that controls the scale of the adjustment in the spring constant for each iteration.

First, from the LEN-binding simulations to the capsid, we picked configurations of LEN complexed to the capsid with monotonically increasing $N_{bound}$. The unbound LEN molecules were removed from the simulation box. The LEN bound to capsid complexes at different values of $N_{bound}$ was then further evolved for 25×10^6^ $\tau _{CG}$. Then, we generated a reduced version of the simulation trajectory by selecting CG site 21 (geometric center) of each CA monomer. Then, the averaged trajectory was computed by aligning all the trajectory snapshots to the initial configuration. We choose a distance cutoff of 5 nm, which typically connects the central CG site with the closest three neighbors (3 $ij$ pairs). The hENM analysis was performed from the final 20×10^6^ $\tau _{CG}$ of the reduced version of the trajectory. The capsid stiffness parameter ($k_{CA- CA}$) is then calculated as the mean harmonic force constant for all $ij$ bonds between CA monomers.

### Live-cell imaging assay

HeLa cells (ATCC CCL-2) and 293T cells (CRL-3216) were obtained from American Type Culture Collection and aliquots of low passage cells were frozen. The low-passage HeLa cells and 293T cells were maintained for a maximum of 1–2 months in Dulbecco’s Modified Eagle’s Medium (DMEM) supplemented with 10% fetal calf serum (Hyclone) and penicillin-streptomycin (50 units/ml and 50 µg/ml, respectively); hereafter referred to as Complete Media. The HeLa cells were tested for mycoplasma contamination using MycoAlert Mycoplasma Detection Kit (Lonza) and MycoStrip Mycoplasma Detection Kit (InvivoGen) and found to be free of mycoplasma using both kits. All cells were maintained in a humidified incubator at 37°C with 5% CO_2_. HeLa:POM121-HALO cells were generated by transduction of HeLa cells with VSV-G-pseudotyped virions containing a lentiviral vector expressing the C-terminal HALO-tagged human POM121. This lentiviral construct was generated by replacing mCherry with HALO in a lentiviral vector that expresses human POM121-mCherry under the control of the ubiquitin C promoter (Burdick et al., 2017).

Virions labeled with GFP-CA and cmHALO were prepared by polyethylenimine transfection (PEI; Polysciences) of 293T cells (3.5×10^6^ cells seeded in 100 mm cell culture dish 1 day prior) with the HIV-1-based vectors pHmNG (40% of the total HIV-1 plasmid amount), pHGFP-GFPCA (10% of the total HIV-1 plasmid amount), and pHmNG-iHALO (50% of the total HIV-1 plasmid amount), along with pHCMV-G (Yee et al., 1994), which expresses the G glycoprotein of vesicular stomatitis virus (VSV-G). pHmNG, which contains an mNeonGreen (mNG) reporter gene in place of *nef* and does not express *env*, was generated by replacing the GFP reporter gene in pHGFP (Burdick et al., 2024) with mNG. pHGFP-GFPCA (Burdick et al., 2020) is similar to pHmNG, except that a GFP-CA fusion protein is generated after proteolytic processing of Gag, and there is a GFP reporter gene in place of *nef*. pHmNG-iHALO expresses free HALO and CA after proteolytic processing, and there is an mNG reporter gene in place of *nef*. To construct pHmNG-iHALO, the GFP in HIV Gag-iGFP ΔEnv (NIH AIDS Reagent Program, Division of AIDS, NIAID, NIH, from Dr. Benjamin Chen; Cat#12455) was replaced with HALO, and then a portion of gag containing HALO was transferred to pHmNG. Three hours after transfection, Complete Media containing 20 nM Janelia Farm 646 (JF646) dye was added to the cells. Unlabeled virions were prepared by PEI transfection of 293T cells with pHmNG and pHCMV-G. Supernatants from the transfected cells were collected 24 hours after transfection, clarified using a 0.45 µm membrane filter, and concentrated by ultracentrifugation (100,000×*g*) for 1.5 hours at 4°C through a 20% sucrose cushion (wt/vol) in 1× Dulbecco’s Phosphate-Buffered Saline with calcium and magnesium (PBS). The concentrated virus was resuspended in 500 µl Complete Media or PBS, and the virus amounts were determined by p24 ELISA (XpressBio).

Viral lysates were prepared using Pierce RIPA buffer (Thermo Fisher) and subjected to western blot analysis using mouse anti-p24 antibody (NIH Aids Reagent Program Cat#24-3), rabbit anti-GFP (Thermo Fisher Cat#A6455), and rabbit anti-HALO (Promega Cat#G9281), followed by IRDye 800CW-labeled goat anti-rabbit secondary antibody (LI-COR Cat #926-32211) or IRDye 680-labeled goat anti-mouse secondary antibody (LI-COR Cat #926-68070). Protein bands were visualized using an Odyssey Infrared Imaging System (LI-COR).

To assess virus infectivity, TZM-bl cells (6×10^3^ cells/well in 96-well plate seeded 1 day prior) were challenged with p24-normalized virus via spinoculation (1200×*g*, 1 hour, 15°C) in the presence of polybrene (10 µg/ml; Sigma). After spinoculation, the cells were incubated at 37°C, and luciferase activity was measured 48 hours after infection using the britelite plus reporter gene assay system (Revvity).

Fluorescent virus particles were analyzed by single virion analysis (Duchon et al., 2024). Virus particles were centrifuged (1200×*g* for 30 min) onto glass-bottom μ-slides (Ibidi) and then imaged using confocal microscopy. GFP and HALO(JF646) signals in each image were detected using Localize (Zenklusen et al., 2008), and the percentage of GFP signals that colocalized with JF646 signal was determined using a custom MATLAB (Mathworks) program.

For live-cell imaging experiments, HeLa:POM121-HALO cells were seeded onto glass-bottom μ-slides (3×10⁴ cells/well) 1 day prior to infection. Before infection, the cells were incubated with Complete Media containing 40 nM JF549 dye and incubated for 30 min at 37°C. The cells were washed twice with Complete Media to remove excess JF549 dye. Next, the cells were challenged with dual-labeled virus via spinoculation (1200×*g* for 1 hour at 15°C) in the presence of polybrene (10 µg/ml) and then incubated at 37°C. At approximately 2 hours post-infection, time-lapse imaging was performed using a Nikon Eclipse Ti-E microscope equipped with a Yokogawa CSU-W1 spinning disk unit and a Plan-Apochromat 100× N.A. 1.49 oil objective. Illumination was provided by 488 nm (GFP), 561 nm (JF549), and 640 nm (JF646) lasers. Confocal z-stacks (3 slices at 0.4 µm step interval centered at the equatorial plane) were acquired every minute for 15 min across five fields of view. Images were captured using a 565 nm splitter and two ORCA-fusion BT cameras (Hamamatsu) and analyzed with Nikon Elements or ImageJ (Briggs et al., 2004). Complete Media containing 2X LEN (or DMSO) was added between the first and second frames, yielding a final LEN concentration of 100 nM. The GFP-CA-labeled particles that remained at the nuclear envelope during the entire 15 min observation period were analyzed. For display purposes, a pixel-averaging filter was applied to the images, and contrast was adjusted.

## Data Availability

The initial system coordinates, input files, force field files, software used for all simulations and summary of the directory content is also available in the https://doi.org/10.5281/zenodo.20098000 public repository. The following dataset was generated: ArpaH 2026Lenacapavir-induced Lattice Hyperstabilization is Central to HIV-1 Capsid Failure at the Nuclear Pore Complex and in the CytoplasmZenodo10.5281/zenodo.20098000PMC1334563342417517

## Additional files

MDAR checklist
