# Supplementary material for: Lenacapavir-induced lattice hyperstabilization is central to HIV-1 capsid failure at the nuclear pore complex and in the cytoplasm
Source: eLife. 2026 Jul 8;14:RP109282. doi: 10.7554/eLife.109282 (PMC13345633; doi:10.7554/eLife.109282)
Supplement: MDAR checklist [file elife-109282-mdarchecklist1.docx]

**Materials Design Analysis Reporting (MDAR)**

**Checklist for Authors**

The [MDAR framework](https://osf.io/xfpn4/) establishes a minimum set of requirements in transparent reporting mainly applicable to studies in the life sciences.

*eLife* asks authors to **provide detailed information within their article** to facilitate the interpretation and replication of their work. Authors can also upload supporting materials to comply with relevant reporting guidelines for health-related research (see [EQUATOR Network](http://www.equator-network.org/%20)), life science research (see the [BioSharing Information Resource](http://biosharing.org/)), or animal research (see the [ARRIVE Guidelines](http://www.plosbiology.org/article/info:doi/10.1371/journal.pbio.1000412) and the [STRANGE Framework](https://doi.org/10.1038/d41586-020-01751-5); for details, see *eLife*’s [Journal Policies](https://reviewer.elifesciences.org/author-guide/journal-policies)). Where applicable, authors should refer to any relevant reporting standards materials in this form.

For all that apply, please note **where in the article** the information is provided. Please note that we also collect information about data availability and ethics in the submission form.

**Materials:**

| **Newly created materials** | **Indicate where provided: section/figure legend** | **N/A** |
| --- | --- | --- |
| The manuscript includes a dedicated "materials availability statement" providing transparent disclosure about availability of newly created materials including details on how materials can be accessed and describing any restrictions on access. | Materials and Methods ("Live-cell imaging assay") and "Data, Materials, and Software Availability". Newly generated plasmids (pHmNG, pHGFP-GFPCA, pHmNG-iHALO) and the HeLa:POM121-HALO stable cell line are available from the corresponding author(s) on reasonable request. Coarse-grained models, force-field and input files are deposited at Zenodo (DOI: 10.5281/zenodo.20098000). |  |
|  |  |  |
| **Antibodies** | **Indicate where provided: section/figure legend** | **N/A** |
| For commercial reagents, provide supplier name, catalogue number and [RRID](https://scicrunch.org/resources), if available. | Materials and Methods ("Live-cell imaging assay", western blot analysis): mouse anti-p24 (NIH AIDS Reagent Program Cat#24-3); rabbit anti-GFP (ThermoFisher Cat#A6455); rabbit anti-HALO (Promega Cat#G9281); IRDye 800CW goat anti-rabbit (LI-COR Cat#926-32211); IRDye 680 goat anti-mouse (LI-COR Cat#926-68070). |  |
|  |  |  |
| **DNA and RNA sequences** | **Indicate where provided: section/figure legend** | **N/A** |
| Short novel DNA or RNA including primers, probes: Sequences should be included or deposited in a public repository. |  | N/A |
|  |  |  |
| **Cell materials** | **Indicate where provided: section/figure legend** | **N/A** |
| Cell lines: Provide species information, strain. Provide accession number in repository OR supplier name, catalog number, clone number, OR RRID. | Materials and Methods ("Live-cell imaging assay"). Human cell lines: HEK293T (ATCC CRL-3216); HeLa and the derived HeLa:POM121-HALO stable line (generated in this study); TZM-bl. |  |
| Primary cultures: Provide species, strain, sex of origin, genetic modification status. |  | N/A |
|  |  |  |
| **Experimental animals** | **Indicate where provided: section/figure legend** | **N/A** |
| Laboratory animals or Model organisms: Provide species, strain, sex, age, genetic modification status. Provide accession number in repository OR supplier name, catalog number, clone number, OR RRID. |  | N/A |
| Animal observed in or captured from the field: Provide species, sex, and age where possible. |  | N/A |
|  |  |  |
| **Plants and microbes** | **Indicate where provided: section/figure legend** | **N/A** |
| Plants: provide species and strain, ecotype and cultivar where relevant, unique accession number if available, and source (including location for collected wild specimens). |  | N/A |
| Microbes: provide species and strain, unique accession number if available, and source. |  | N/A |
|  |  |  |
| **Human research participants** | **Indicate where provided: section/figure legend) or state if these demographics were not collected** | **N/A** |
| If collected and within the bounds of privacy constraints report on age, sex, gender and ethnicity for all study participants. |  | N/A |

**Design:**

| **Study protocol** | **Indicate where provided: section/figure legend** | **N/A** |
| --- | --- | --- |
| If the study protocol has been pre-registered, provide DOI. For clinical trials, provide the trial registration number OR cite DOI. |  | N/A |
|  |  |  |
| **Laboratory protocol** | **Indicate where provided: section/figure legend** | **N/A** |
| Provide DOI OR other citation details if detailed step-by-step protocols are available. | Materials and Methods. Step-by-step single-virion analysis protocol is published in Duchon et al., HIV Protocols, Springer US (2024), DOI: 10.1007/978-1-0716-3862-0_6. |  |
|  |  |  |
| **Experimental study design (statistics details) *** | | |
| **For in vivo studies: State whether and how the following have been done** | **Indicate where provided: section/figure legend. If it could have been done, but was not, write “not done”** | **N/A** |
| Sample size determination |  | N/A |
| Randomisation |  | N/A |
| Blinding |  | N/A |
| Inclusion/exclusion criteria | Pre-established inclusion criterion: only GFP-CA-labeled cores that remained stably docked at the nuclear envelope for the entire 15-minute observation period were analyzed (Methods, "Live-cell imaging assay"; Fig 4). |  |
|  |  |  |
| **Sample definition and in-laboratory replication** | **Indicate where provided: section/figure legend** | **N/A** |
| State number of times the experiment was replicated in the laboratory. | Methods and figure legends. CG MD: two independent NPC-docking replicas (Fig 2, 3); four independent well-tempered metadynamics replicas for free capsids (Fig 5). Live-cell imaging: independent cells/cores pooled from five fields of view (Fig 4C: 237 DMSO and 192 LEN cells; Fig 4D: 29 and 37 cores). |  |
| Define whether data describe technical or biological replicates. |  | N/A |
|  |  |  |
| **Ethics** | **Indicate where provided: section/submission form** | **N/A** |
| Studies involving human participants: State details of authority granting ethics approval (IRB or equivalent committee(s), provide reference number for approval. |  | N/A |
| Studies involving experimental animals: State details of authority granting ethics approval (IRB or equivalent committee(s), provide reference number for approval. |  | N/A |
| Studies involving specimen and field samples: State if relevant permits obtained, provide details of authority approving study; if none were required, explain why. |  | N/A |
|  |  |  |
| **Dual Use Research of Concern (DURC)** | **Indicate where provided: section/submission form** | **N/A** |
| If study is subject to dual use research of concern regulations, state the authority granting approval and reference number for the regulatory approval. |  | N/A |

**Analysis:**

| **Attrition** | **Indicate where provided: section/figure legend** | **N/A** |
| --- | --- | --- |
| Describe whether exclusion criteria were pre-established. Report if sample or data points were omitted from analysis. If yes, report if this was due to attrition or intentional exclusion and provide justification. | Methods ("Live-cell imaging assay"). Inclusion/exclusion criteria were pre-established (only cores stably docked at the nuclear envelope for the full 15-min window were analyzed). No data points were omitted. |  |
|  |  |  |
| **Statistics** | **Indicate where provided: section/figure legend** | **N/A** |
| Describe statistical tests used and justify choice of tests. | Methods and figure legends. Live-cell categorical comparisons were analyzed by Fisher’s exact test (Fig 4C-F; ns, P > 0.05). Simulation observables are reported as mean ± standard deviation across independent replicas (Fig 2, 3, 5 and supplements); virion colocalization is reported as AVG ± SD (Fig S4E). |  |
|  |  |  |
| **Data availability** | **Indicate where provided: section/submission form** | **N/A** |
| For newly created and reused datasets, the manuscript includes a data availability statement that provides details for access (or notes restrictions on access). | "Data, Materials, and Software Availability" section. Initial system coordinates, input files and force-field files are publicly available at Zenodo, DOI: 10.5281/zenodo.20098000. |  |
| When newly created datasets are publicly available, provide accession number in repository OR DOI and licensing details where available. | Publicly available at Zenodo, DOI: 10.5281/zenodo.20098000. |  |
| If reused data is publicly available provide accession number in repository OR DOI, OR URL, OR citation. | Reused structural data cited in Methods: LEN-CA hexamer X-ray structure PDB 6VKV (Bester et al. 2020); human NPC cryo-ET model (Mosalaganti et al. 2022); HIV-1 capsid CG model (Hudait et al. 2024). |  |
|  |  |  |
| **Code availability** | **Indicate where provided: section/figure legend** | **N/A** |
| For any computer code/software/mathematical algorithms essential for replicating the main findings of the study, whether newly generated or re-used, the manuscript includes a data availability statement that provides details for access or notes restrictions. | "Data, Materials, and Software Availability" section. Simulation input files, force fields and analysis software are publicly available at Zenodo, DOI: 10.5281/zenodo.20098000. |  |
| Where newly generated code is publicly available, provide accession number in repository, OR DOI OR URL and licensing details where available. State any restrictions on code availability or accessibility. | Newly generated LAMMPS input scripts, force-field files and analysis code are deposited at Zenodo, DOI: 10.5281/zenodo.20098000 (no access restrictions). |  |
| If reused code is publicly available provide accession number in repository OR DOI OR URL, OR citation. | Reused public software cited in Methods: LAMMPS (Plimpton 1995); VMD (Humphrey et al. 1996); well-tempered metadynamics (Barducci et al. 2008). |  |

**Reporting:**

The MDAR framework recommends adoption of discipline-specific guidelines, established and endorsed through community initiatives.

| **Adherence to community standards** | **Indicate where provided: section/figure legend** | **N/A** |
| --- | --- | --- |
| State if relevant guidelines (e.g., ICMJE, MIBBI, ARRIVE, STRANGE) have been followed, and whether a checklist (e.g., CONSORT, PRISMA, ARRIVE) is provided with the manuscript. |  | N/A |
