# Supplementary material for: Lenacapavir-induced lattice hyperstabilization is central to HIV-1 capsid failure at the nuclear pore complex and in the cytoplasm
Source: eLife. 2026 Jul 8;14:RP109282. doi: 10.7554/eLife.109282 (PMC13345633; doi:10.7554/eLife.109282)
Supplement: Peer review history

# Editor report: eLife Assessment

**DOI:** 10.7554/eLife.109282.4.sa0

**Authors:** Bin Zhang

**Affiliations:** Massachusetts Institute of Technology United States

**Keywords:** Convincing, Important

This study investigates how the HIV inhibitor lenacapavir influences capsid mechanics and interactions with the nuclear pore complex. It provides **important** insights into how drug-induced hyperstabilization of the viral shell can compromise its structural integrity during nuclear entry. The modeling is technically sophisticated, and the analyses provide **convincing** support for the mechanistic conclusions.

# Referee report: Reviewer #1 (Public review)

**DOI:** 10.7554/eLife.109282.4.sa1

[Editors' note: this version has been assessed by the Reviewing Editor without further input from the original reviewers. The authors have addressed the comments raised in the previous round of review.]

The paper from Hudait and Voth details a number of coarse-grained simulations as well as some experiments focused on the stability of HIV capsids in the presence of the drug lenacapavir. The authors find that LEN hyperstabilizes the capsid, making it fragile and prone to breaking inside the nuclear pore complex.

Comments on previous round of revisions:

I found that the authors addressed my concerns satisfactorily. The other reviewer raised a number of important points regarding the nuances of the model and the interpretation of the simulations, which the authors rebutted. I think the paper in its current form now is a worthwhile addition to the literature.

# Referee report: Reviewer #3 (Public review)

**DOI:** 10.7554/eLife.109282.4.sa2

This is a technically sophisticated study that integrates coarse-grained modeling with live-cell imaging to address an important and timely question regarding HIV-1 capsid inhibition by lenacapavir.

In summary, in my view, the manuscript represents a solid contribution to the field.

# Author comment: Author response

**DOI:** 10.7554/eLife.109282.4.sa3

**Authors:** Arpa Hudait, Ryan C Burdick, Ellie K Bare, Vinay K Pathak, Gregory A Voth

**Affiliations:** University of Chicago Chicago United States; National Cancer Institute Center for Cancer Research Frederick United States; National Cancer Institute Center for Cancer Research Frederick United States; National Cancer Institute Center for Cancer Research Frederick United States; University of Chicago Chicago United States

The following is the authors’ response to the previous reviews

**Public Reviews:**

**Reviewer #1 (Public review):**

The paper from Hudait and Voth details a number of coarse-grained simulations as well as some experiments focused on the stability of HIV capsids in the presence of the drug lenacapavir. The authors find that LEN hyperstabilizes the capsid, making it fragile and prone to breaking inside the nuclear pore complex.

I found the paper interesting. I have a few suggestions for clarification and/or improvement.

(1) How directly comparable are the NPC-capsid and capsid-only simulations? A major result rests on the conclusion that the kinetics of rupture are faster inside the NPC, but are the numbers of LENs bound identical? Is the time really comparable, given that the simulations have different starting points? I'm not really doubting the result, but I think it could be made more rigorous/quantitative.

(2) Related to the above, it is stated on page 12 that, based on the estimated free-energy barrier, pentamer dissociation should occur in ~10 us of CG time. But certainly, the simulations cover at least this length of time?

(3) At first, I was surprised that even in a CG simulation, LEN would spontaneously bind to the correct site. But if I read the SI correctly, LEN was parameterized specifically to bind to hexamers and not pentamers. This is fine, but I think it's worth describing in the main text.

Comments on revisions:

I found that the authors addressed my concerns satisfactorily. The other reviewer raised a number of important points regarding the nuances of the model and the interpretation of the simulations, which the authors rebutted. I think the paper in its current form now is a worthwhile addition to the literature.

**Reviewer #3 (Public review):**

I have carefully reviewed the manuscript, the two referee reports, and the authors' detailed responses. I appreciate the substantial effort the authors have invested in addressing the reviewers' comments, and I also recognize the strength and ambition of the work. This is a technically sophisticated study that integrates coarse-grained modeling with live-cell imaging to address an important and timely question regarding HIV-1 capsid inhibition by lenacapavir.

Embedded within Reviewer #2's report are several substantive points that warrant careful consideration, particularly with respect to framing, terminology, and engagement with the broader literature. I view my role here is to distinguish those issues from claims that I do not find to be supported.

We thank Reviewer 3 for the positive assessment of our work.

First, I do not agree with Reviewer #2's central assertion that the manuscript lacks novelty or fails to present meaningful new findings. While individual elements of the system studied herecapsid docking at the NPC, lenacapavir-induced capsid hyperstabilization, capsid rupture, and competition with FG- nucleoporins-have been observed previously, this work provides a coherent, mechanistic account of how these elements are coupled. In particular, the proposed sequence linking LEN-induced lattice hyperstabilization, preferential pentamer loss at the narrow end, NPC-induced mechanical stress, and failure of nuclear import represents a nontrivial integration that goes beyond prior phenomenological observations. I therefore do not view this work as redundant with existing literature.

We thank Reviewer 3 for the positive assessment of our work.

That said, Reviewer #2 is correct to note that the manuscript would benefit from broader and more explicit engagement with recent independent studies, including computational and hybrid modeling efforts that address capsid mechanics, nuclear entry, and LEN effects using different frameworks. While the authors' bottom-up coarse-grained approach is clearly distinct and, in many respects, more systematically derived, eLife readers would benefit from a clearer discussion of how the present results relate to, complement, or differ from these other approaches. I strongly encourage the authors to add a short discussion paragraph situating their work within this broader context, without disparaging alternative models.

We have now added several sentences describing papers that use two other CG models that are of some relevance to our work at the beginning of the fourth paragraph of the Introduction, and we have also highlighted the distinguishing features of our work at the end of that paragraph.

Second, I find that some mechanistic claims in the manuscript would benefit from more careful language distinguishing model-conditioned interpretation from de novo prediction. This applies in particular to discussions of LEN binding heterogeneity and stoichiometry, as well as to conclusions drawn from biased enhanced-sampling simulations. While I agree with the authors that parameterization does not invalidate mechanistic insight, it is important to be precise about what aspects of the behavior emerge from the simulations versus what is constrained by prior experimental knowledge. Modest tightening/revising of language (e.g., "suggests," "is consistent with," "within the model") would address this concern without weakening the scientific conclusions.

We have revised and softened the language in several places as suggested. However, we do still asert that our overall CG modeling approach is quite rigorous. The use of limited “top down” information on LEN binding is not problematic and in fact warranted in this problem.

Third, Reviewer #2 raises a legitimate semantic issue regarding the use of the term "elasticity." The manuscript infers changes in capsid mechanical response using heterogeneous elastic network models, which quantify effective stiffness and deformability rather than elasticity in the macroscopic materials sense. I recommend that the authors clarify this definition explicitly in the text to avoid confusion and unnecessary debate.

We have now added a clarification at the end of the third paragraph of the subsection entitled “LEN binding to the capsid results in hyperstabilized lattice domains”. We have also added text in the second paragraph of the Discussion. Our view is that our perspective is more useful for this problem than a “macroscopic” perspective as the capsid is, in fact, a mesoscopic object and not a macroscopic one.

Finally, I note that several of Reviewer #2's objections-particularly those asserting circular reasoning, misuse of enhanced sampling methods, or invalidity of coarse-grained predictions reflect a misunderstanding of contemporary bottom-up coarse-grained modeling rather than genuine methodological flaws. I do not believe these points require further rebuttal or revision beyond what the authors have already provided.

We agree.

In summary, in my view, the manuscript represents a solid contribution to the field, provided that the authors undertake a limited set of targeted revisions aimed at improving framing, clarity, and engagement with the broader literature. Addressing these points will strengthen the manuscript and ensure that its contributions are clearly and fairly communicated to the community.

We have done so as suggested by the reviewer.
